# The repertoire of short tandem repeats across the tree of life

**DOI:** 10.1186/s13059-025-03893-z

**Published:** 2025-12-12

**Authors:** Nikol Chantzi, Ilias Georgakopoulos-Soares

**Affiliations:** Division of Pharmacology and Toxicology, College of Pharmacy, The University of Texas at Austin, Dell Pediatric Research Institute, Austin, TX USA

## Abstract

**Background:**

Short tandem repeats (STRs) are widespread, dynamic repetitive elements with a number of biological functions and relevance to human diseases, genome plasticity and adaptation. However, their prevalence across taxa remains poorly characterized.

**Results:**

Here, we examined the prevalence and distribution of STRs across the complete genomes of 117,861 organisms spanning the tree of life. We find that there are large differences in the frequencies of STRs between organismal genomes, and these differences are largely driven by the taxonomic group an organism belongs to. Using simulated genomes, we find that on average, there is no enrichment of STRs in bacterial and archaeal genomes, suggesting that these genomes are not particularly repetitive. In contrast, we find that eukaryotic genomes are orders of magnitude more repetitive than expected. STRs are preferentially located at functional loci in specific taxa. Finally, we utilize the recently completed Telomere-to-Telomere genomes of human and other great apes, and find that STRs are highly abundant and variable between primate species, particularly in peri/centromeric regions.

**Conclusions:**

We conclude that STRs have expanded in eukaryotic and viral lineages and not in archaea or bacteria, resulting in large discrepancies in genomic composition.

**Supplementary Information:**

The online version contains supplementary material available at 10.1186/s13059-025-03893-z.

## Introduction

The rapid decline in the cost of DNA sequencing has facilitated the generation of complete genomes for an ever-growing number of organisms across the tree of life. A number of large, international consortia are currently underway with the goal of sequencing genomes representing the diversity found in nature [1–3]. This offers a remarkable opportunity to increase our understanding of genome architecture, evolution, and diversity in unprecedented depth and breadth. Additionally, the recent completion of the human genome through the Telomere-to-Telomere (T2T) Consortium [4] enables the examination of the distribution and frequency of repeat elements in regions of the genome that were only partially annotated previously, including centromeres and telomeres. Another recent effort includes the T2T-Primates consortium [5], which has completed the generation of diploid assemblies for multiple non-human primate species and through which we can gain insights into the diversity, evolution, and plasticity of different repeats in the primate lineage.

Repetitive DNA sequences are widespread in the genomes of various life forms. For example, over half of the human genome is composed of repetitive sequences [6]. Nevertheless, we still do not fully understand the landscape of repeat elements in organisms across the tree of life. Initially thought of as junk DNA, repeat elements are fast evolving, and are usually classified into two major classes, tandem repeats and dispersed repeats, the second of which also encompasses transposable elements [7]. Short tandem repeats (STRs) are defined as multiple consecutive copies of an oligonucleotide repeat unit, often defined for up to 1–9 base-pairs (bp) unit length [8]. These are highly polymorphic, representing a major source of genetic variation, due to their heightened mutation rate [9].

In the human genome, there are more than one million tandem repeats [10], which vary substantially in the population [11–13]. STRs are mutational hotspots, associated with genomic instability [14], providing a source for genomic diversity, polymorphism, and organismal adaptation, while they are also linked to a number of human diseases [10, 15]. STRs trigger slippage events during DNA replication, leading to frequent mutations in the number of repeat units, through insertions and deletions [16]. As a result of the genomic instability associated with them, STRs are causative for a number of human diseases [15], including Mendelian disorders [17], neurodegenerative disorders [10], and contribute to multiple complex traits and phenotypes [10, 18]. In addition, large STR expansions have been identified in different cancer types [19], and, in microorganisms, STRs are also known to be related to pathogenicity and genomic variability [20, 21].

Furthermore, STRs can form non-canonical DNA structures such as Z-DNA, G-quadruplexes and hairpins, which are also associated with a higher mutation rate and have a plethora of functional roles [22–26]. STRs have been linked to a number of functions, including roles in gene regulation [27–30], recombination [31, 32], epigenetic changes [33], and genome architecture [34] among others. The instability of STRs has various implications and applications in molecular biology. They enable DNA forensics, PCR-based genotyping and phenotyping, fine-scale phylogenetic analysis, paternity testing, linkage-disequilibrium mapping, and hitchhiking mapping [35, 36]. In eukaryotic organisms, STRs are also highly prevalent in pericentromeric and centromeric regions [37, 38]. However, systematic study of these regions has been challenging due to the limitations of short-read sequencing technologies, which struggle to accurately determine the sequence composition of these parts of the genome [39].

Because of their higher mutation and turnover rates, STRs are of extreme interest in understanding evolution. STRs play a crucial role in driving species evolution and contribute significantly to the diversity and adaptability of life forms [40]. Multiple studies have investigated the prevalence of STRs in specific taxonomic groups [41–46]. Nevertheless, despite their importance, there is currently no comprehensive analysis of the distribution, diversity and topography of STRs among taxa spanning the different domains, kingdoms and phyla in the tree of life.

Here we perform the largest comprehensive examination of tandem repeat variations across 117,861 complete organismal genomes spanning all major taxonomies of the tree of life. We find that STRs are most enriched in eukaryotic organisms, with *Plasmodium falciparum* exhibiting the highest STR density. Using genome simulation experiments, we find that on average archaea and bacteria show a relative depletion of STRs, whereas eukaryotes show the highest STR enrichment (roughly 10-fold), relative to what is expected by chance. We find large differences in the frequency of STRs with different repeat unit lengths, which are also influenced by the taxonomic group and the genomic compartment. Finally, we perform an in-depth analysis of the recently published T2T primate genomes. We find marked enrichment of STRs in pericentromeric and centromeric regions, which have rapidly and dynamically evolved in primate genomes. These enrichments are driven by specific STR units and tend to be dinucleotide and pentanucleotide. Surprisingly, large differences in the STR composition, frequency, and location are also observed between chromosomes in these genomes. These findings indicate large differences in the frequency and topography across the tree of life, acting as a highly dynamic and polymorphic genomic element.

## Results

### Systematic identification of short tandem repeats in organismal genomes

We examined 117,861 complete organismal genomes spanning the tree of life for the genome-wide prevalence of short tandem repeats (STRs), consisting of 49,192 bacterial, 47,842 viral, 687 archaeal, and 490 eukaryotic genomes, respectively (Table S1−2). STRs were defined as consecutive genomic segments of at least 10 base pairs long, for unit lengths between one and nine base-pairs (bp). We identified STR sequences across the complete genomes of each available organism, creating a comprehensive genome-wide dataset with species from diverse taxa across the tree of life. In total, we identified 82,593,090 STRs, of at least 10 base pairs long, across the 117,861 complete organismal genomes. We observed that the average STR density was 1.51 STR bp per kB (Fig S1). However, we find that there are large differences depending on the organism and its taxonomic group. Across all the examined species, we observe that *Plasmodium falciparum* shows the highest STR genomic density (109 bp/kB), with a 73-fold enrichment over the average STR density across organismal genomes, and 11.32-fold enrichment over the average STR density across eukaryotes. Additionally, we report that 19,645 viruses and 5 bacteria lacked STRs altogether (Fig S1). This is consistent with a previous work studying 719 organismal genomes, in which *Plasmodium falciparum* was also found to have the highest STR density [41] and consistent with it having a highly repetitive, AT-rich genome [47].

**Fig. 1:**
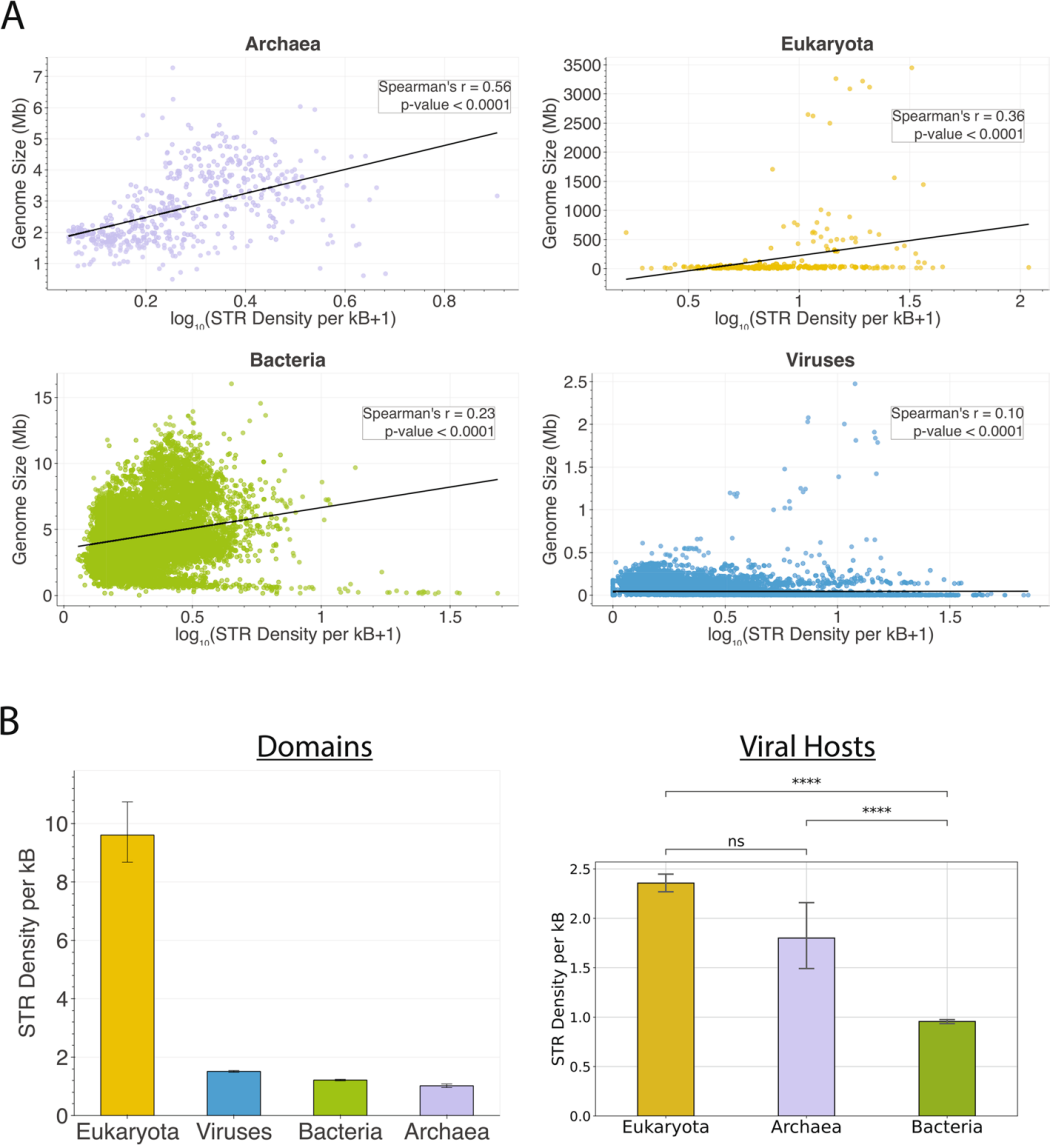
Characterization of STRs in 117,861 organismal genomes across the three domains of life and viruses.** A** Association between the genome size and the proportion of the genome covered by STRs, presented separately for the three domains of life and viruses. **B** Average STR bp density per kB for organisms in the three domains of life and viruses and for viruses relative to the viral host domain. Error bars represent 95% bootstrap confidence intervals. Statistical significance was assessed using a two-tailed independent t-test, and *p*-values were adjusted for multiple hypothesis testing using the Benjamini-Hochberg procedure. Adjusted p-values are displayed as * for *p* < 0.05, ** for *p* < 0.01, *** for *p* < 0.001, and **** for *p* < 0.0001

Next, we investigated if there is a correlation between the genome size and the density of STRs in each of the three domains of life and viruses. We find that archaea and eukaryotes exhibit the highest correlations (Spearman correlations of *R* = 0.56 and *R* = 0.36), whereas bacteria and viruses show weaker correlations (Spearman correlations of *R* = 0.23 and *R* = 0.10) (Fig. 1a). When comparing the domains of life, the highest genomic density of STRs was observed in eukaryotes with 9.60 STR bp per kB, whereas the lowest was observed in archaea with 1.02 STR bp per kB (Fig. 1b). For viruses, we further subdivided the analysis by their host type and found that viruses of eukaryotic hosts had the highest genomic density of STRs, with both eukaryotic and archaeal hosts displaying significantly higher STR density than bacterial hosts (Fig. 1b; two-tailed independent t-test, *p*-value < 0.0001). These findings indicate that STRs are most abundant in eukaryotic genomes, in which their frequency correlates with genome size.

### Characterization of tandem repeat frequencies across taxa

**Fig. 2:**
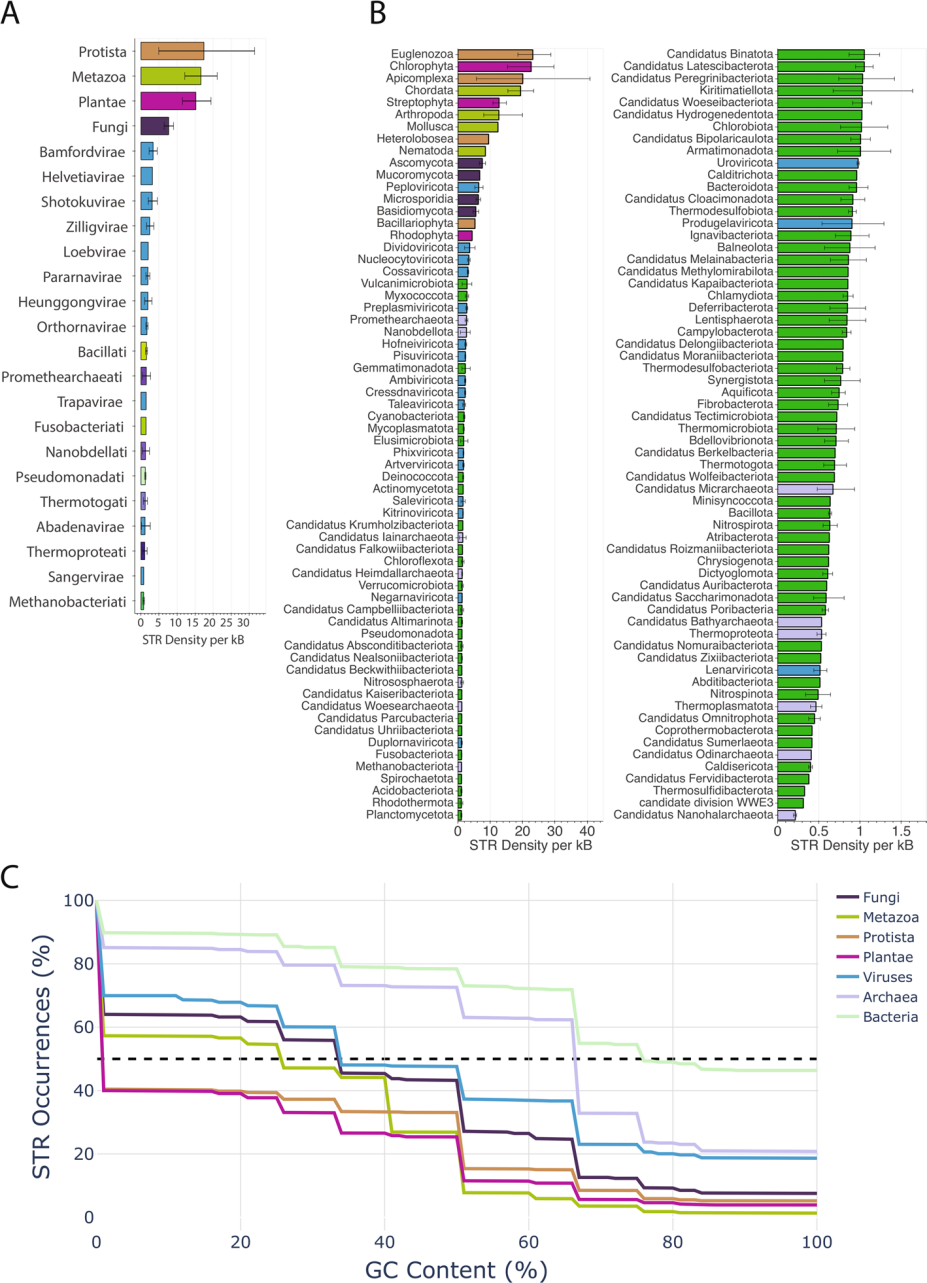
Taxonomic characterization of STRs across the tree of life.** A** Density of STRs in each kingdom. Error bars show 95% bootstrap confidence intervals. Coloring is performed at the domain of life level. **B** Density of STRs in each phylum. Error bars show 95% bootstrap confidence intervals. Coloring is performed at the kingdom level. **C** STR occurrences as a function of the STR GC content in the STR sequences. Results shown at the kingdom and domain level

Next, we examined differences in the distribution and density of STRs between taxonomic subgroups. We observe that at the kingdom level, Protista, Metazoa, Plantae, and Fungi, all of which are eukaryotic, show the highest STR density, with Protista having an average of 21,45 STR bp per kB. This is followed by different viral kingdoms, whereas the archaeal kingdom Thermoproteati displays the lowest genomic STR density (Fig. 2a), consistent with our observations at the domain level. We also examined the average STR density across organisms belonging to the same phylum. We find that consistently eukaryotic phyla show the highest STR density, with Euglenozoa and Apicomplexa having, on average, 23.16 and 20.06 STR bp per kB (Fig. 2b).

We investigated the density of STRs detected as a function of GC content across the kingdoms; we observed that by increasing the GC content threshold to 10%, the total STR occurrences in Protista dropped to 455,813 from 1,127,402, which is equivalent to a 59.57% reduction in STR motifs (Fig. 2c). The drop in STR occurrences detected as a function of GC content is sharper in eukaryotic organisms with a reduction of 35.96%, 42.70%, and 60.00% in Fungi, Metazoa, and Plantae kingdoms, respectively, in contrast to Archaea, Bacteria, and Viruses (Fig. 2c; Fig S2-3). Additionally, when examining the relationship of STR density and the genome-wide GC content, we report that in Eukaryota the GC content is negatively correlated with STR density (Spearman correlation *r*=−0.27, p-value < 0.0001), whereas prokaryotes are positively correlated (Spearman correlation *r* = 0.49 and *r* = 0.41, p-value < 0.0001 for Bacteria and Archaea, respectively). However, viral genomes do not exhibit such correlation (Spearman *r*=−0.01, p-value > 0.05) (Fig S2-3). These results can be explained by the higher Adenosine/Thymine (AT) content of eukaryotic genomes, which is reflected in higher AT content in STRs. We conclude that the frequency of short tandem repeats varies significantly across taxa across the domain, kingdom, and phylum levels.

### Significant variation in the abundance of different STR repeat units in different taxa

**Fig. 3:**
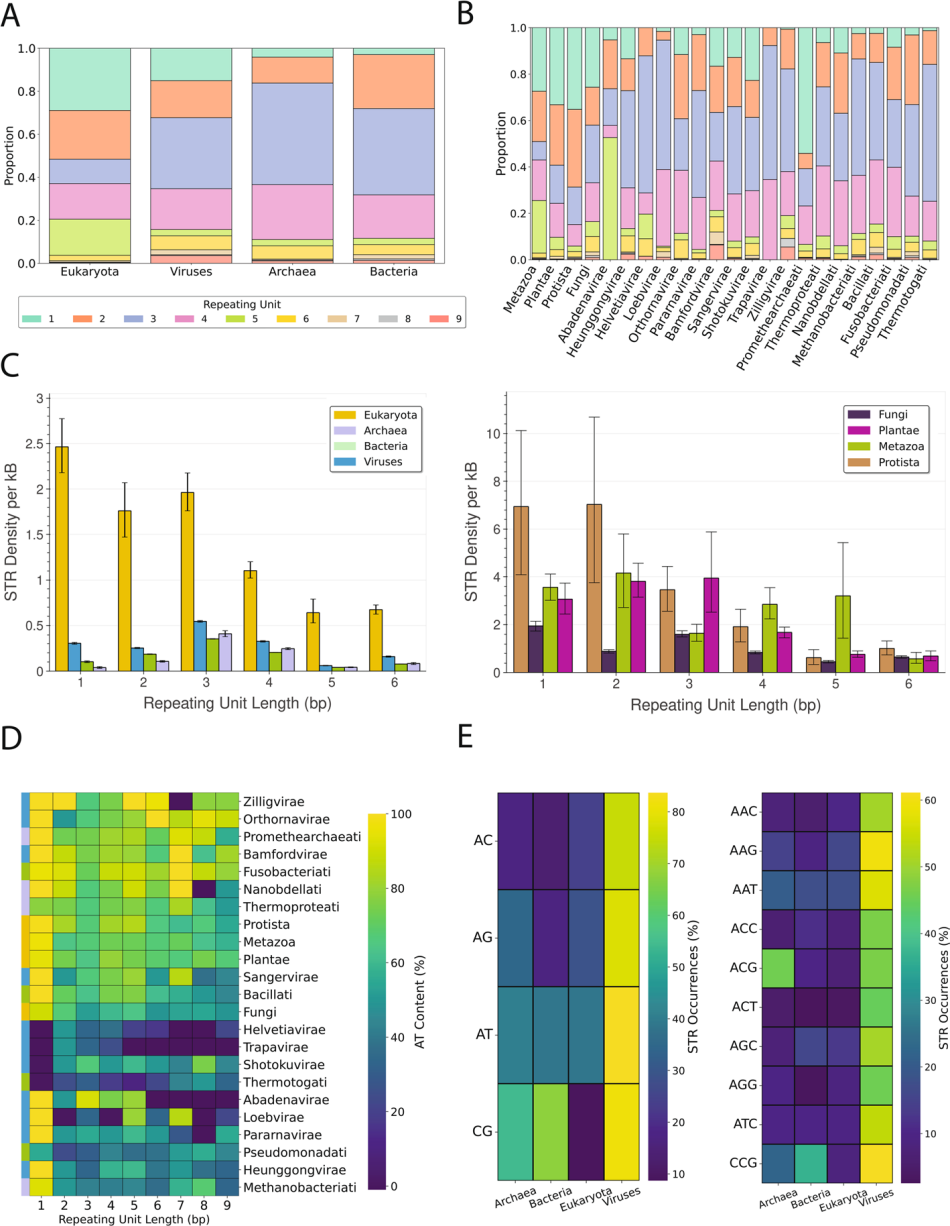
STR density for different repeat unit lengths and nucleotide composition in organisms across the tree of life.** A** Proportion of total STR occurrences stratified by the repeating unit length for the three domains of life and viruses. **B** Proportion of total STR occurrences stratified by the repeating unit length per kingdom. **C** Average density of STRs stratified by the repeating unit length per domain of life and kingdom. **D** Percentage of AT Content of STRs stratified by the repeating unit length and kingdom. **E** Empirical mean of the proportion of total dinucleotide (left) and trinucleotide (right) STRs partitioned by domain of life, including viruses. STRs, their reverse complements, and their cyclic shifts were considered identical. The representative dinucleotide and trinucleotide consensus motif was defined as the minimum in the lexicographical order

To investigate potential differences among taxonomic groups based on the most common types of STRs in their genomes, we categorized the STRs according to the length of the repeat unit. We observe that in Bacteria, Archaea, and Viruses trinucleotide STRs are the most abundant, whereas in Plantae, Protista, Fungi and Metazoa mononucleotide STRs are most abundant (Fig. 3a-b). Of interest is also the fact that in animals, when calculated as total base pairs, the first most prevalent STR type is pentanucleotide STRs (Fig. 3b), which could be driven by telomeric and centromeric regions.

We found that in eukaryotes the most frequent STRs are those with 1 bp repeat unit length, whereas in prokaryotic and viral genomes the most frequent are those of 3 bp repeat unit length (Fig. 3c; Fig S4). This can be explained by the larger proportion of the genomic space being coding in prokaryotic and viral genomes, in which trinucleotide repeats maintain the reading frame in contrast to mono and dinucleotide repeat units. When partitioning the organismal genomes into the different kingdoms, we find in all eukaryotic kingdoms, mononucleotide STRs are the most frequent. Notably, in Protista and Metazoa the second most frequent is dinucleotide STRs, whereas in Plantae trinucleotide STRs. We also observe a non-negligible density of tetranucleotide and pentanucleotide STR exclusive in animals (Fig. 3c; Fig S5). Using the available T2T genomes, we observe that certain species display long stretches of STRs spanning hundreds of thousands of base pairs, particularly in both animal and plant genomes (Table S3). Notably, some of these long STRs correspond to known telomeric repeats or their cyclic permutations, such as ccctaa*/ttaggg* in animals and gggttta*/aacccta* in plants [48, 49]. For example, in *Ziziphus jujuba*, we detect a cyclic variant of the plant telomeric repeat gggttta. These findings indicate variation in the frequency of the different STR unit lengths between taxa.

**Fig. 4:**
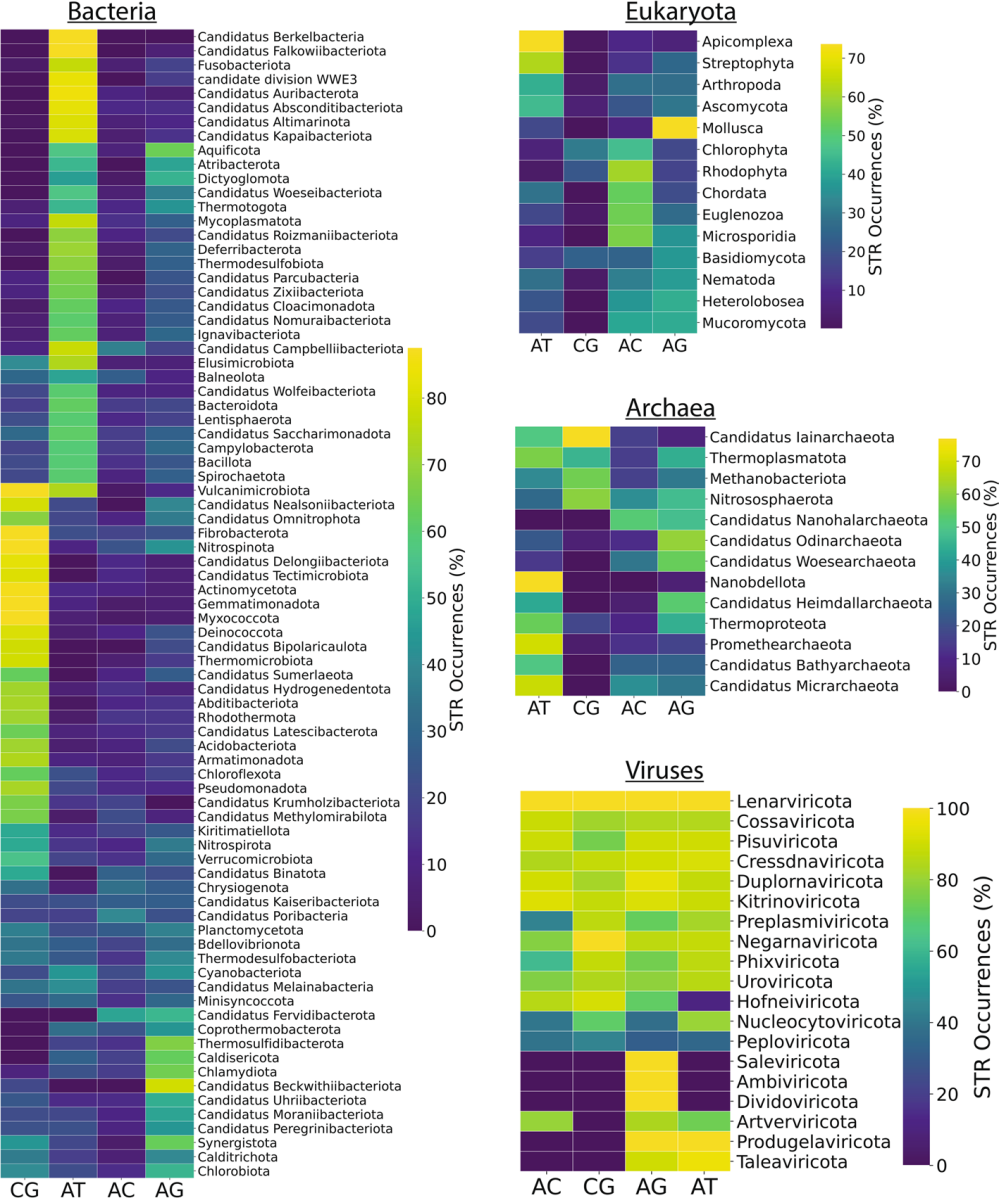
Prevalence of dinucleotide STRs across phyla in the domains of life and viruses. Hierarchical clustering of dinucleotide STRs. The values represent the empirical mean of the occurrences (%) of each STR motif within each phylum. Results are shown separately for phyla belonging to each domain of life and in viruses. Dinucleotide consensus motifs of STRs, their reverse complements, and their cyclic shifts were considered identical. The representative dinucleotide consensus motif was defined as the minimum in the lexicographical order

Next, we investigated the AT composition of STRs. We stratified organismal genomes by their kingdoms and calculated the median of the total percentage of AT content in the STRs across each species. We observed that mononucleotide repeats are predominantly AT-driven in Viruses, Protista, Metazoa and Plantae kingdoms (Fig. 3d). Notably, the septanucleotide STRs in Viruses are also highly AT-driven (Fig. 3d). Upon closer examination, we report that the septanucleotide repeats tatttta, aataatt, consist of 52% and 30%, respectively, of the total septanucleotide repeats found in viral genomes, with the overwhelming majority (> 98%) emerging within the *Orthopoxvirus monkeypox* genome of the Bamfordvirae kingdom. Upon examining the dinucleotide repeats, the GC and CG tandem repeats were the most frequent dinucleotide repeat type in Bacteria, whereas in eukaryotic organismal genomes, the differences were more sparsely scattered, with TA, AT as well as GT and CA sharing a large proportion of the existing STRs, indicating that there are significant underlying differences between the various kingdoms (Fig. 3e). Finally, trinucleotide STRs revealed a more equally dispersed distribution, with a notable exception of trinucleotide ACG in Archaea, which are highly enriched (Fig. 3e). These differences suggested that the analysis could be conducted on the phylum level. Upon further partitioning of the genomes in phyla, we observed that GC-rich STRs are highly specific to a distinct collection of bacterial phyla, whereas other STRs are predominantly AT rich or more uniformly distributed (Fig. 4). Furthermore, in eukaryotic phyla, CA dinucleotide repeats seem to emerge more frequently than the other dinucleotide STR categories, and TA/AT constitute the vast majority of Apicomplexa STRs (Fig. 4). Similar results were observed for trinucleotide STRs, with marked differences being observed both at the domain and phylum levels. For instance, the strong trinucleotide ACG signal in Archaea originates exclusively from 255 distinct Methanobacteriota species, and it is the most frequent trinucleotide STR motif, occurring in total 34,501 times (Fig S6-7). Overall, the biophysical properties of STRs are highly divergent across the different domains of life and kingdoms and different STR patterns emerge that are highly specific, indicating that they are involved in different biological mechanisms and molecular processes depending on the organism.

### STRs are enriched in eukaryotic and viral, but not in bacterial or archaeal genomes

**Fig. 5:**
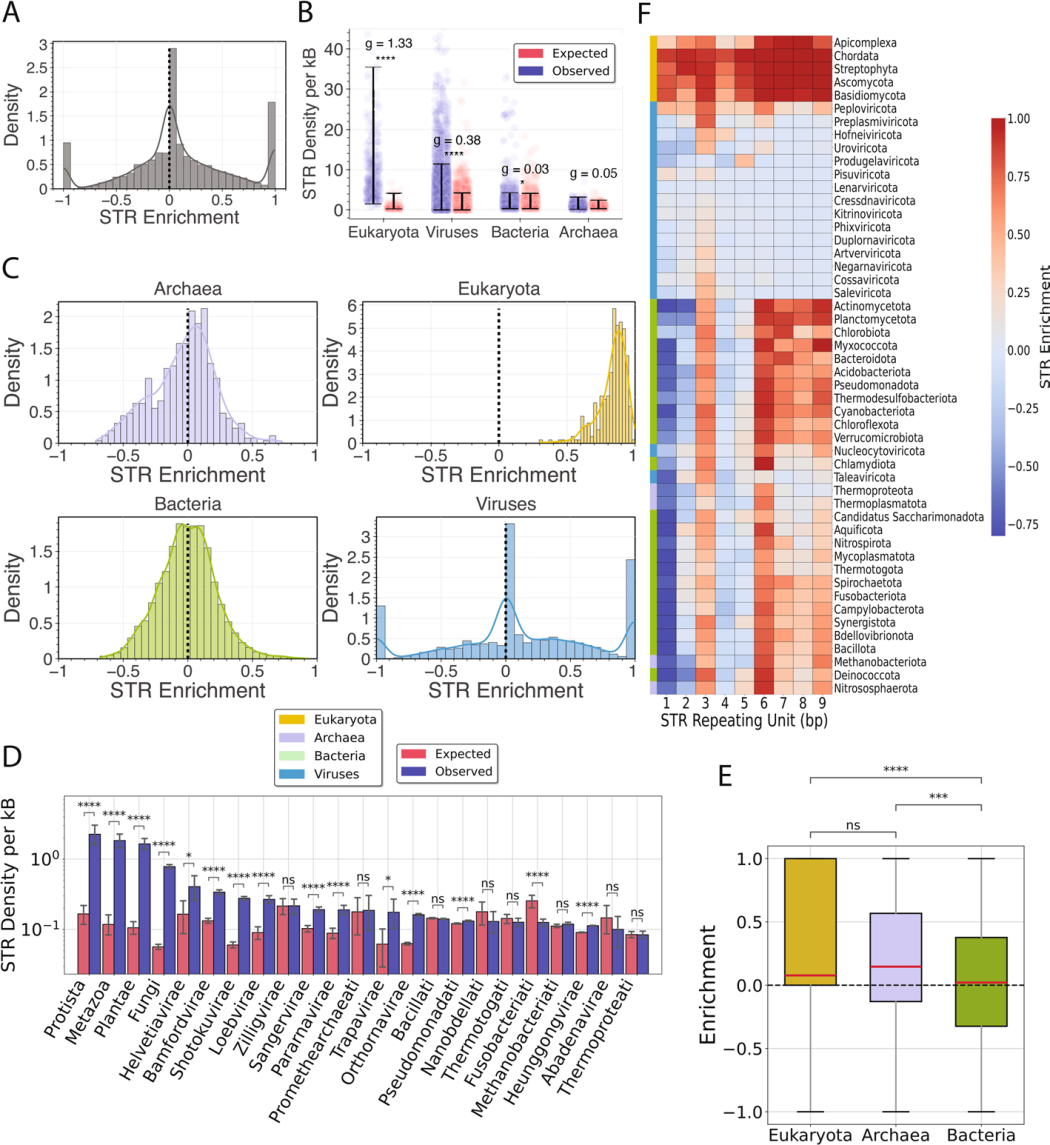
Enrichment of STRs in organismal genomes. **A** Distribution of STR enrichment across organismal genomes. Histogram shows the density of probability of STR enrichment across organismal genomes relative to matched controls; the overlaid curve is the kernel density estimate. STR enrichment was calculated as (O − E)/(O + E), where O is the total STR base pairs and E is the total base pairs in matched control regions. **B** Jitter plots show the genome-wide STR density for each species across Eukaryota, Bacteria, Archaea, and Viruses, compared with their matched shuffled-genome expectation. The y-axis is capped at 45 STR per kB. Error bars denote the 97.5% quantile-based confidence interval. The g represents Hedges’ effect size. Two-tailed independent t-tests were performed and adjusted for multiple comparisons using Benjamini-Hochberg procedure. **C** Distribution of STR enrichment across organismal genomes stratified by superkingdom. Histogram shows the density of probability of STR enrichment across organismal genomes relative to matched controls; the overlaid curve is the kernel density estimate. **D** STR density comparison between organismal genomes and the control group, partitioned by kingdom. The performed tests are two-tailed independent t-tests, adjusted for multiple comparisons using Benjamini-Hochberg procedure. **E** STR enrichment in viral genomes, separated by their host domain of life. The performed tests are two-tailed independent t-tests, adjusted for multiple comparisons using Benjamini-Hochberg procedure. **F** Clustermap of STR enrichment in organismal genomes across the different kingdoms. The STR enrichment was calculated as the ratio of the difference between the observed and the expected STR base pairs to the total number of STR base pairs found both in the original and the shuffled genome (see methods). Adjusted p-values are displayed as * for *p* < 0.05, ** for *p* < 0.01, *** for *p* < 0.001, and **** for *p* < 0.0001

We next examined if STRs are found more frequently in organismal genomes than expected by chance. To that end, for every organismal genome we generated a matched simulated genome [50], controlling for dinucleotide content, resulting in 117,861 simulated genomes. We quantified the density of different STR categories across taxonomies, at the domain, kingdom, and phylum levels. To address the question of the prevalence of STRs in a given organismal genome, we compared the resulting STR densities in the real and the shuffled genomes. When examining the three domains of life and viruses, we find that eukaryotes and viruses have, on average, a 13.76-fold and 2.35-fold enrichment of STRs than expected by chance (two-tailed t-test independent, *p*-value < 0.0001). In contrast, bacteria and archaea show, on average, a 1.02-fold and 0.99-fold enrichment, respectively, with negligible effect sizes (Hedges’ *g* = 0.03 and 0.05*)* indicating neither enrichment nor depletion of STRs on the domain level (Fig. 5a-b).

Interestingly, we observe that all eukaryotic species are enriched in STRs, indicating that this is universally the case (Fig. 5b). Additionally, in bacteria, archaea, and viruses there is a significant amount of variance amongst the various organismal genomes (Fig. 5b). Furthermore, in several cases, viral genomes that were initially found not to contain any STRs, in the simulated genomes, contained several STRs and thus obtained a maximum enrichment score of 1.00. Analogously, initially viral genomes that had STRs, after the chromosomal permutations, were rendered empty due to stochastic effects, obtaining the minimum enrichment score of −1.00 (Fig. 5c). This phenomenon was exclusive to viruses, due to inherently smaller genome sizes of viral genomes, which allowed for such extreme cases to occur. Among eukaryotes, the largest enrichments were observed in Protists (enrichment = 1.0), Fungi (enrichment = 0.96), followed by Plantae (enrichment = 0.96), and Metazoa (enrichment = 0.96) (Fig. 5d; Fig S8). Upon partitioning the various domains into kingdoms, we notice that that the bacterial kingdom Fusobacteriati is depleted of STRs (Fig. 5c; two-tailed independent t-test with Benjamini-Hochberg correction for multiple comparisons; p-value < 0.0001). When separating viruses by their host in those infecting eukaryotes, archaea, and bacteria, we observe that eukaryotic viruses (2.38 STRs per kB) and archaeal viruses (1.61 STRs per kB) have a higher STR density than bacterial viruses (0.91 STRs per kB) viruses (two-tailed independent t-test, p-value < 0.0001), but archaeal and eukaryotic viruses do not display statistically significant differences (two-tailed independent t-test, p-value > 0.05). Additionally, archaeal viruses show a significantly higher STR enrichment than bacterial viruses (two-tailed independent t-test, p-value < 0.0001), the latter of which have the lowest STR enrichment, with the STR density being similar to that of the simulated genomes (Fig. 5e).

Due to the high variance amongst viral and prokaryotic genomes, we examined how the enrichment of STRs varies in the various phyla, for all repeating unit lengths. We observe a significant amount of enrichment for all eukaryotic phyla, and for all repeat unit lengths. However, for prokaryotic genomes, we notice that one bp repeat unit STRs are highly depleted. Notably, trinucleotide and hexanucleotide tandem repeats are also enriched in most prokaryotic phyla, but not in several viral phyla (Fig. 5f; Fig S8, two-tailed t-test independent with Benjamini-Hochberg correction for multiple comparisons, *p*-value < 0.05). This is in contrast with eukaryotes, in which one bp repeat unit STRs are highly enriched (Fig. 5f). Interestingly, there is also a subset of STRs of repeat unit length of six to nine bp that are highly enriched in prokaryotic phyla (Fig. 5f). These findings indicate that bacterial and archaeal genomes are, on average, not more repetitive than expected by chance in contrast to eukaryotic and viral genomes.

### STRs are preferentially positioned relative to functional elements in specific taxa

**Fig. 6:**
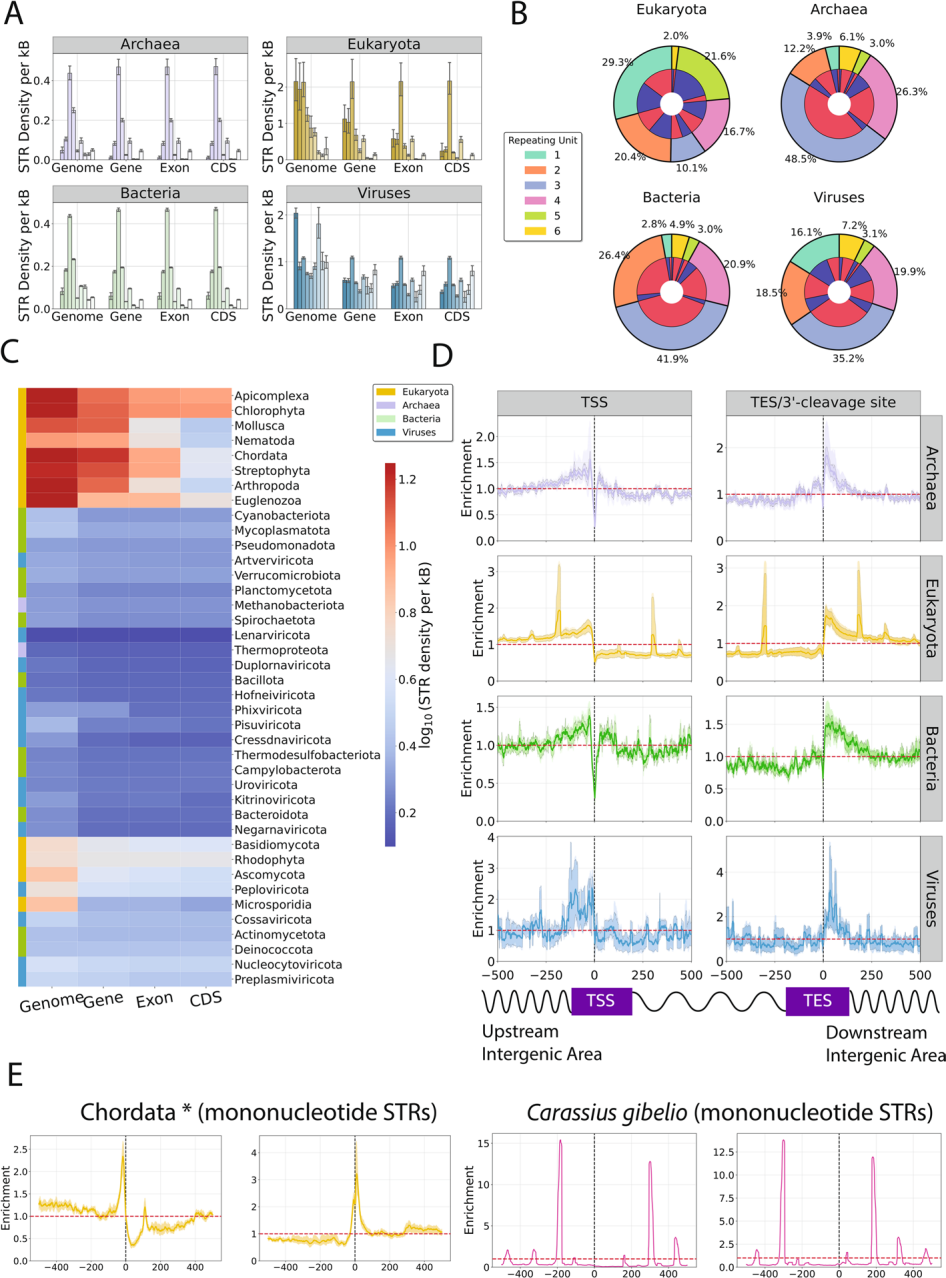
The topography of STRs relative to genomic subcompartments and transcription/translation start and translation end/3’-cleavage sites, across taxa. **A** STR density for the three domains of life and viruses at the genome, genic, exonic, and coding regions. **B** Pie charts showing the percentage of STRs separated by STR unit length and proportion being genic and intergenic for the three domains of life and viruses. **C** STR density of organisms belonging to different phyla at the genome, genic, exonic, and coding regions. **D** STR distribution across the three domains of life and viruses, relative to the translation/transcription start sites (TSS), translation end site (TES)/3’-cleavage sites. In many RNA viruses, gene boundaries mark open reading frames, translation start and stop sites, rather than transcriptional units. **E** Mononucleotide STR distribution in chordate (excluding Carassius gibelio) and in Carassius gibelio. Confidence intervals represent the 2.5% lowest and 97.5% highest percentiles from Monte-Carlo simulations with replacement on the species (inner layer) and on the family (outer layer) taxonomic level (*N* = 1,000)

Next, we investigated the distribution of STRs across different genomic elements, including genic, exonic and CDS regions. At the phylum level, for the vast majority of phyla, the STR density is highest when looking on the genome level, rather than any specific genome subcompartment, indicating that STRs are enriched in intergenic areas (Fig. 6a-b). This pattern is consistent across unit lengths, with only exceptions being trinucleotide and hexanucleotide STRs, which are more enriched in genic, exonic and CDS regions (Fig. 6a-c). For Chordata, Streptophyta, Chlorophyta, and Apicomplexa, we observe high STR densities in genic and exonic regions. In particular, for Chlorophyta and Apicomplexa, we observe high STR densities in CDS regions, besides genic and exonic (Fig. 6c). In prokaryotic genomes, we observe a general depletion of STRs, with several notable exceptions (Fig. 6c). For instance, the bacterial phyla Deinococcota and Bacteroidota display a high genome-wide STR density and similarly high genic/CDS density of STRs. We also observe that trinucleotide unit STRs are enriched across these compartments, which is explained by the selection against disrupting reading frames [45, 46, 51].

### Enrichment of STRs relative to transcription start and end sites

Provided that the distribution of STRs was found to be uneven across genomic subcompartments, we investigated the distribution of STRs relative to functional genomic elements, including Transcription Start Sites (TSSs) and Transcription End Sites (TESs). Our hypothesis was that if STRs have functional roles, they will be enriched relative to key genomic loci. We find that viruses are enriched upstream of the TSS and downstream of the TES. In many RNA viruses, annotated gene boundaries correspond to open reading frames, i.e., translation start and stop sites, rather than discrete transcriptional units. In bacteria and archaea weaker effects were observed, with weak enrichments found upstream and downstream of genic regions (Fig. 6 d; Fig S9). Eukaryotes showed strong and highly localized enrichment at roughly 150 bp upstream from the TSS, 300 bp downstream from the TSS, and similarly roughly 300 bp upstream of the TES and 150 bp downstream of the TES (Fig. 6 d; Fig S9).

Nonetheless, around the modes, the confidence intervals were highly volatile, suggesting that the observed signal originated from a certain subset of species and putatively a STR of specific length. This led us to separate the eukaryotic phyla, partition the distributions across the various repeating unit lengths of STRs and examine separately the STR enrichment relative to the TSS and TES, finding significant differences between them. Examination of the density of mononucleotide STRs amongst Chordata revealed that this sharp enrichment stemmed from mononucleotide STRs in *Carassius gibelio*, whereas the other members of the Chordata displayed an enrichment at the TSS and TES (Fig. 6e). Subsequent examination of the remaining eukaryotic phyla showed that Ascomycota and Apicomplexa have polyA and polyT mononucleotide STRs enriched directly at the TSS and TES. However, Streptophyta mononucleotide repeat distribution was comparable to Chordata, but the polyA repeats were, instead, replaced with polyT repeats (Fig S10). 

### Large differences in STR frequency across and within human chromosomes

Using the T2T complete human genome [4], we examined the distribution, frequency, and diversity of STRs across the complete chromosomes, including low complexity regions such as pericentromeric, centromeric, and telomeric loci, which have been traditionally difficult to accurately sequence and study [52].

**Fig. 7:**
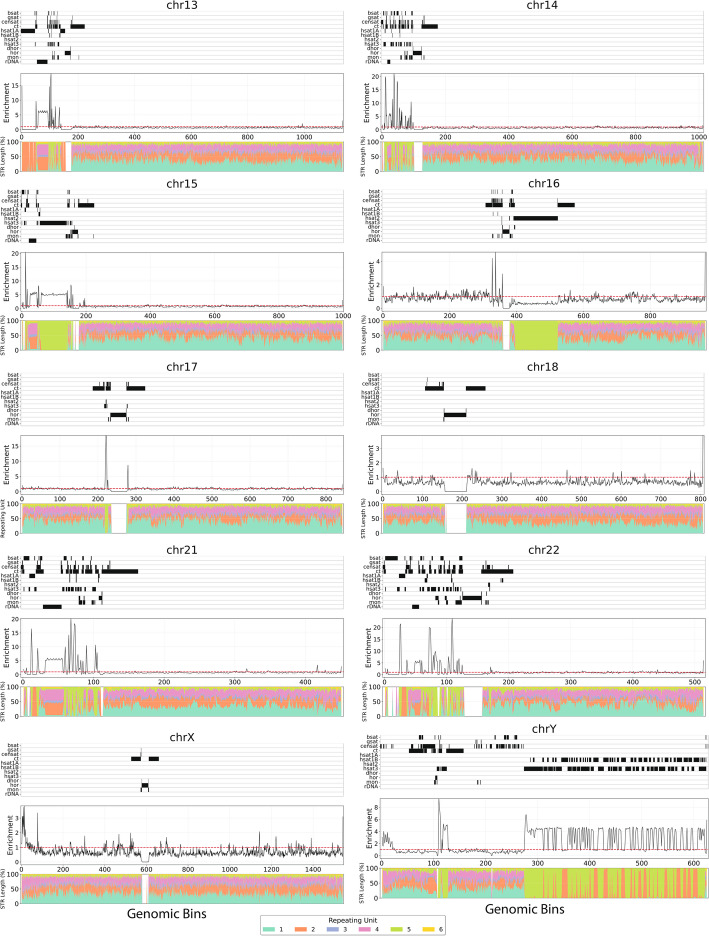
Characterization of STRs across chromosomes in the T2T reference human genome, each one equipartitioned into mutually exclusive regions of 100 kb length. Schematics show the distribution of STRs across different human chromosomes. The top panel aligns the position of centromeric and pericentromeric regions, with blackcolor representing the presence of the repeat in that genomic region. Line plots show the STR fold enrichment at each genomic 100 kb length bin for a chromosome. Stacked barplots show the results for different STR repeating unit lengths. Repeats include inactive αSat HOR (hor), divergent αSat HOR (dhor), monomeric αSat (mon), classical human satellite 1 A (hsat1A), classical human satellite 1B (hsat1B), classical human satellite 2 (hsat2), classical human satellite 3 (hsat3), beta satellite (bsat), gamma satellite (gsat), other centromeric satellites (censat) and centromeric transition regions (ct)

In total we report 3,642,514 STRs, of at least 10 bp long, in the T2T human genome. We investigated the STR composition and density across and within each chromosome. To examine the relationship between STRs and centromeres, each chromosome was annotated by the relevant pericentromeric and centromeric positions. We report marked differences in STR distribution patterns between and within chromosomes. Strong STR enrichments are observed in chromosomes 21 and 22, in the short chromosome arms. Particularly striking is the cluster of STRs in the short arm of chromosome 21 (21p) at ribosomal DNA (rDNA) genes. In contrast, chromosomes 1, 2, 3, 4, 5, 7, 10, 16, 17, and 20 show sharp STR peaks in the vicinity of the centromeres and in pericentromeric regions (Fig. 7; Fig S11-12). For chromosomes 6, 8, 11, 12, 18, and 19 we do not find strong STR enrichments at any part of the chromosome (Fig. 7; Fig S12).

In the acrocentric chromosomes 13, 14, and 15, the HSat3 arrays span the centromere, displaying strong enrichment on the short arm. Interestingly, in the aforementioned chromosomes, the rDNA array genomic region, which is proximal to the centromere, shows a particularly strong STR enrichment, with the exception that its corresponding STR composition is highly diverse (Fig S13); a phenomenon which is in contrast to other highly repetitive STR-enriched regions where the STRs are biased towards a specific repeat unit length. Interestingly, chromosome 9 is an outlier, with the whole centromere being composed of five bp unit STR repeats, with this region displaying the highest enrichment in the chromosome. The Y chromosome displays a strong STR enrichment in over half of its length, which are composed primarily of dinucleotide and pentanucleotide STRs (Fig. 7). Amplicons are highly prevalent in mammalian Y chromosomes [53] and account for the repeated pattern of STR enrichment with intervals over long genomic distances (Fig. 7). These results indicate the highly differing STR profiles of the different human chromosomes, while acrocentric chromosomes show a distinct and consistent pattern of high STR density in the shortest arm.

**Fig. 8:**
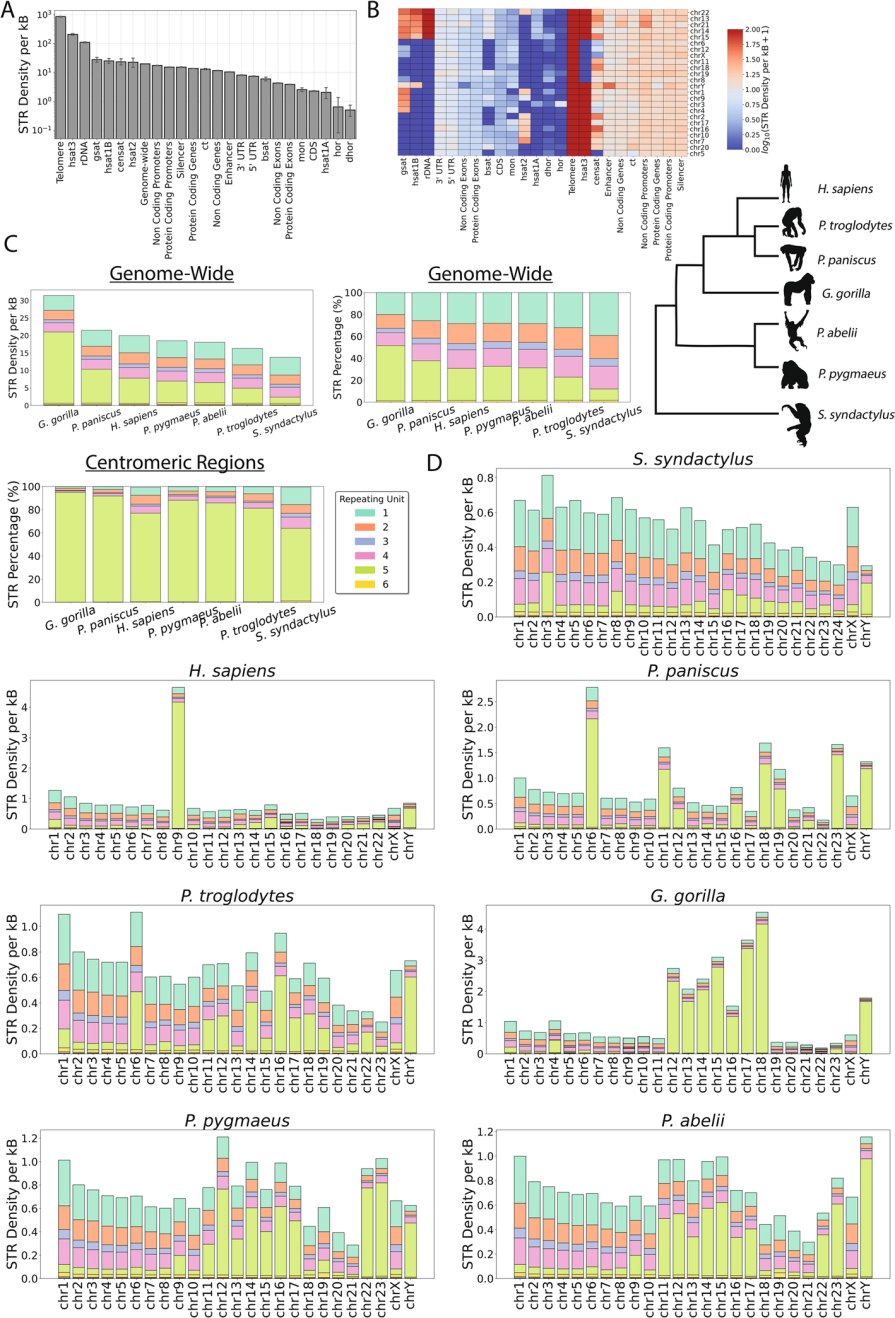
Distribution of STRs in Telomere-to-Telomere primate genomes.** A** STR Density across human genome sub-compartments including centromeric repeats. **B** STR Density across human genome sub-compartments including centromeric repeats split by chromosome. **C** Percentage and density of different lengths of STRs genome-wide and in centromeres. **D** STR density for STRs of different unit length across primate species, in each of their chromosomes. Repeats include inactive αSat HOR (hor), divergent αSat HOR (dhor), monomeric αSat (mon), classical human satellite 1 A (hsat1A), classical human satellite 1B (hsat1B), classical human satellite 2 (hsat2), classical human satellite 3 (hsat3), beta satellite (bsat), gamma satellite (gsat), other centromeric satellites (censat) and centromeric transition regions (ct). A simplified primate phylogenetic schematic was added for context (based on [55])

Next, we investigated the STR density across human genomic subcompartments, including the different types of satellite array regions that are part of the pericentric and centromeric repeats. We find that STRs are most enriched in telomeric regions (Fig. 8a), which is consistent with them being composed of STRs [54]. These are followed by classical human satellite 3 (hsat3) repeats and rDNA loci (Fig. 8a). Interestingly, silencers and inactive and divergent alpha satellite higher-order repeats show the strongest depletion for STRs (Fig. 8b). When examining each repeat length separately, we find that hexanucleotide STRs are most prevalent in telomeres, which is expected, and STRs of five bp repeat unit length are most prevalent in classical human satellite 2 (hsat2), hsat3 repeats. In contrast, hsat1A and hsat1B sites contain mostly dinucleotide STRs and gamma satellites (gsat) mononucleotide STRs) (Fig. 8b).

### T2T primate genomes reveal rapid and dynamic evolution of STRs in the primate lineage

Next, we used the genomes from the T2T-Primates consortium [5], and investigated the distribution of STRs in six non-human primate genomes including *Gorilla gorilla* (gorilla), *Pan paniscus* (bonobo), *Pan troglodytes* (chimpanzee), *Pongo abelii* (Sumatran orangutan), *Pongo pygmaeus* (Bornean orangutan) and *Symphalangus syndactylus* (Siamang gibbon). Among primates, we find that *Gorilla gorilla* shows the highest STR genome-wide density, whereas *Symphalangus syndactylus* has the lowest genome-wide STR density (Fig. 8b).

Centromeres have arisen during eukaryotic evolution that are some of the most fast evolving regions of the genome and therefore were particularly interested to examine the STR diversity and distribution in centromeric and pericentromeric repeats across the primate species. We also find that in multiple primate species, including humans, gorilla and bonobo, pentanucleotide STRs are the most abundant type of STR (Fig. 8b). In repeats found in centromeric and pericentromeric regions, pentanucleotide STRs are the most abundant type (Fig. 8b; Fig S12). In contrast, in repeats found in telomeric regions, hexanucleotide STRs are the most abundant type (Fig S14).

Recent work has indicated that the sex chromosomes are highly repetitive and has examined the presence of long palindromes, endogenous repeat elements, and satellites [56–58]. However, the contribution of STR repeat types has not been examined. Here, we focused on the distribution of STRs throughout the sex chromosomes in the primate lineage. Interestingly, the STR profile of chromosome Y is remarkably different between the human and non-human primate genomes (Figs. 7 and 8, Fig S15). In contrast, the STR content of chromosome X is markedly similar between these genomes (Fig. 8, Fig S16).

### STR density predicts indel burden across Pseudomonadota

**Fig. 9:**
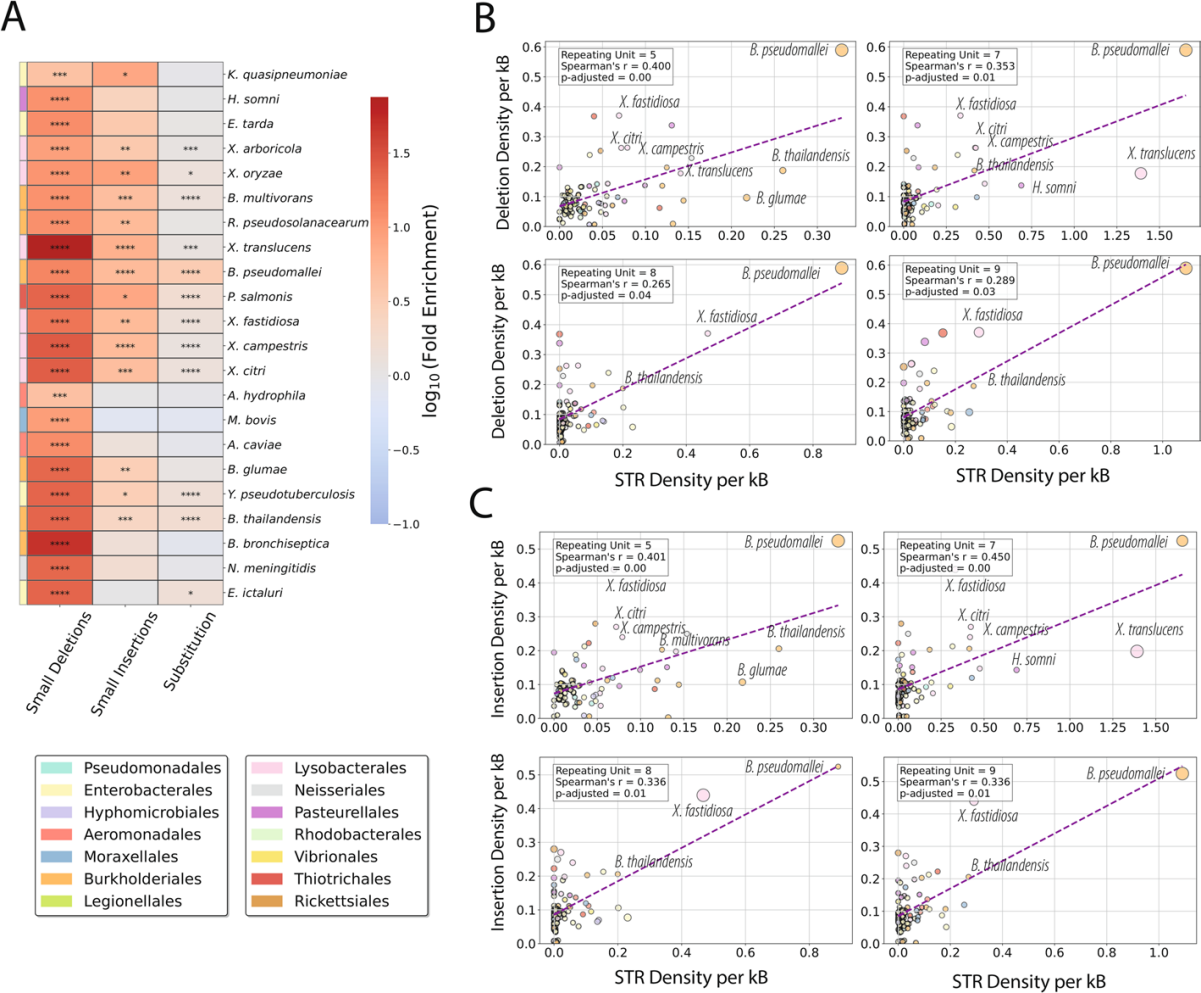
Association of interspecies polymorphic loci with STRs across bacterial organisms.** a** Log fold enrichment of proportion of polymorphic loci overlapping with at least one STR overlapping polymorphic sites compared to matched controls across 87 bacterial species. Statistical significance was assessed using a one-tailed Fisher’s exact test, and p-values were adjusted for multiple hypothesis testing using the Benjamini–Hochberg procedure. Adjusted p-values are displayed as * for *p* < 0.05, ** for *p* < 0.01, *** for *p* < 0.001, and **** for *p* < 0.0001. **b** STR density for repeating units 5, 7, 8, and, 9 regressed against small insertion polymorphic density for each mutational type across 87 bacterial species, with bubble sizes representing the influence of outliers on the regression as measured by Cook’s distance. **c** STR density for repeating units 5, 7, 8, and, 9 regressed against small deletion polymorphic density for each mutational type across 87 bacterial species, with bubble sizes representing the influence of outliers on the regression as measured by Cook’s distance

Our simulations between shuffled genomes displayed substantial differences in the STR distribution amongst the bacteria phyla. The distribution exhibited skewness, with many bacterial phyla being depleted of STRs. To test how STR density varies with repeat-unit length at polymorphic loci, we catalogued intraspecies variants across 87 Pseudomonadota species (see Methods). Overall, we find strong enrichment for substitutions, small insertions and deletions across most species, with deletions showing the strongest effect (Fig. 9a). We report significant positive correlations between STR density and the densities of small insertions and deletions for repeat-unit lengths 5, 7, 8, and 9, with the strongest signal in pentanucleotide repeats (Fig. 9b, c; Fig S17) (Spearman’s correlation *r* = 0.4, *r* = 0.41, respectively, adjusted p-value < 0.0001). This is consistent with the slipped-strand mispairing mechanism [59], which constitutes a hallmark of STR mutagenic etiology. Interestingly, several bacterial pathogens emerged as strong outliers that disproportionately influenced the regression. Specifically, we distinguished *Burkholderia pseudomallei*, a sacrophylic, opportunistic pathogen, known for the induction of melioidosis [60], which was the most influential outlier in the regression of STR density against small deletions and small insertions for various repeating unit lengths (Fig. 9b, c). This species exhibited the highest rates of small deletions and insertions, with 9.8% of its total STRs coinciding with small deletions. These observations support the notion that while prokaryotic genomes are generally depleted of STRs compared to eukaryotic genomes, a subset of bacterial pathogens show significant STR accumulation [61]. Specifically, when compared against matched controls, we observe that STRs are enriched at indels across bacterial species (Fig. 9B; one-tailed Fisher’s exact test, p-values adjusted for multiple comparisons). Collectively, these findings indicate that STRs are pervasive, lineage-modulated hotspots of indel mutagenesis in bacteria whose skewed distribution and enrichment at polymorphic sites likely shape bacterial genome plasticity and adaptive evolution.

## Discussion

In this study, we conducted a comprehensive analysis of STR variations across 117,861 organismal genomes from a broad range of organisms spanning all major taxonomic groups. Our findings reveal that STRs are predominantly enriched in eukaryotic organisms, with the highest STR density observed in *Plasmodium falciparum*. We found an extremely high variance between domains, with eukaryotic organisms having the highest STR density and archaea the lowest. Interestingly, similar variation in STR density was identified upon partitioning the viruses with respect to the host domain. Viruses of eukaryotic hosts had a disproportionately higher STR density than the corresponding viruses of bacterial and archaeal hosts. *O*n the kingdom level, Protista had the highest average STR density, followed by Metazoa. Upon further partitioning on the phylum level, Euglenozoa had the highest average STR density followed by several other eukaryotic phyla. The bacterial and archaeal genomes have fewer intergenic regions and most prokaryotic phyla showed low STR densities, with some notable exceptions, in accordance with previous literature [21, 46, 62].

STRs are drivers of genome evolution and adaptation [40, 61], are highly dynamic elements and can expand or contract primarily due to strand-slippage replication events. Therefore, slippage events can result in the addition or deletion of repeat units, leading to insertions or deletions. These mutations can have various effects, ranging from benign to harmful, depending on their location and contribute to human genetic disorders, especially those characterized by repeat expansions, such as Huntington’s disease and muscular dystrophy [13]. Thus, understanding how different organisms cope with the genomic instability is of importance. Through genome simulation experiments, we discovered that archaea and bacteria typically exhibit fewer STRs than expected, while eukaryotes possess a roughly ten-fold enrichment. When separating viruses by their host domain, eukaryotic viruses show higher STR enrichment than archaeal and bacterial viruses. We also observed that STRs tend to be located near functional genomic elements in specific taxa. These results highlight substantial variations in the frequency and distribution of STRs across the tree of life. It should be noted that there were considerable differences in enrichment of STRs across the various phyla, potentially indicating the STRs have evolved to assist different functionalities and biological mechanisms in different phyla or that they are more likely to expand in certain phyla than others. These results are consistent with the current literature, where STRs have an uneven distribution in prokaryotic genomes [61].

Purifying selection is known to act on STR loci preventing further expansion [61, 63]. The predominance of A/T mononucleotide repeats across eukaryotes likely reflects multiple forces: smaller effective population sizes (relative to prokaryotes) that weaken purifying selection and allow A/T runs to persist, plus retrotransposition-driven seeding of poly(A)/T tracts, such as LINEs use 3′ poly(A) tails and preferentially integrate at T-rich sites, depositing additional A/T repeats [64, 65]. Additionally, pentanucleotide repeats within primate genomes are associated with centromeric architecture and hexanucleotide repeats are involved with telomeric maintenance, showcasing the roles of STRs in genome architecture. In contrast, STRs are not enriched in prokaryotic genomes; nevertheless there is a subset of bacterial genomes that utilize STR-related mechanisms for rapid adaptation. Previous research has indicated that pathogenic bacteria exhibit long stretches of STRs [61]. For instance, STRs have been hypothesized to be an integral part of the adaptive machinery of *B. pseudomallei*, a highly infectious and antibiotic resistance pathogen [66]. This adaptation is facilitated by increased polymorphism within STR loci which enables bacterial pathogens to regulate genes without substantially increasing the overall mutation rate [67].

The frequency of STRs varies significantly for different repeat unit lengths and is influenced by the organism’s taxonomic classification. Several STR motifs were far more prevalent when focusing on a specific domain. For instance, dinucleotide STRs in bacteria, most commonly emerge with the consensus GC or CG. In general, STRs in prokaryotes were GC-enriched in contrast to other eukaryotic or viral species, due to the genome GC content differences. The depletion of trinucleotide STRs in animals likely reflects their low coding fraction and the stringent purifying selection on frame-preserving repeats in exons, which together compress the genomic niche where 3 bp repeats can persist. We also examined if STRs are preferentially located at certain genomic compartments such as genic, exonic and CDS regions. By partitioning STRs into unique sets of one to nine bp unit length, we observed that across all prokaryotic organisms, trinucleotide repeats are the most prevalent in CDS regions, which is expected for maintaining the reading frame in coding regions. In several eukaryotic and viral species, the genic density of STRs was higher than the corresponding CDS density, leading us to the conclusion that STRs have a higher density in intronic and intergenic rather than exonic areas. Furthermore, mononucleotide repeats constituted 29.4% of total STRs in eukaryotic species, followed by 21.6% of pentanucleotide STRs and 20.4% of dinucleotide STRs. Dinucleotide and pentanucleotide STRs were concentrated in non-genic areas, as indicated in the human genome and non-human primate genomes, in which they were highly enriched in centromeric and pericentromeric regions.

The distribution of STRs relative to TSSs, TESs and 3’-cleavage sites was also highly variable depending on the taxon. Most interestingly, in eukaryotic species, an enrichment relative to the TSS and TES led us to segment further into phyla and individual species in our analysis. To our surprise, the signal originated from a goldfish, which had an enrichment of mononucleotide repeats roughly 150 bp upstream from the TSS. Various protists and fungi phyla displayed enrichment of STRs relative to the TSS, with mononucleotide and dinucleotide repeat density reaching the highest enrichment just before the TSS and just after the 3’-cleavage site. This is in contrast to other eukaryotic genomes originating from animal or plant phyla, such as Streptophyta or Chordata, where the transcriptional regulatory roles of STRs were far more diverse with several positions relative to transcription start and end sites being enriched. Proximal to the TSS, mononucleotide repeats on the non-template strand in Chordata are predominantly polyA repeats, whereas in Streptophyta, they are mostly polyT repeats. This difference may reflect an evolutionary divergence, as Streptophyta appear to have replaced A-rich polyadenylation signals with T-rich motifs [68], distinguishing them from other eukaryotic phyla. In bacteria and archaea, STRs were preferentially positioned downstream of the TESs. These results suggest that STRs repeats play a crucial role in various underlying biological mechanisms but their regulatory roles vary greatly across the different phylum, kingdom and domain taxonomic levels.

Finally, we use the recently completed T2T genomes of various primate species, including the latest assembly of the human genome, to discover that STRs are highly abundant and variable between primate species, especially in peri/centromeric regions. Our findings indicate that STRs are highly dynamic, fast-evolving elements facilitating organismal evolution. The vast differences observed at Y chromosomes amongst the Great Apes and Homo Sapiens are suggestive of their intrinsic plastic nature. The Y chromosome in *Homo Sapiens* is covered by HSat1-3 arrays which account for most of its part. On Y chromosome satellite arrays HSat2-3 being enriched in pentanucleotide repeats whereas hsat1A and hsat1B are composed of dinucleotide repeats is a common feature amongst primates. The observed differences on the Y chromosome could be attributed to the tendency of heterochromatin to be highly mutagenic with observed differences even amongst homologous chromosomes. Such differences are not observed in the X chromosomes, which STR are more uniformly distributed and far more diverse for the various repeating unit lengths. In such regions, due to the highly repetitive nature of STR-rich loci, double-strand breaks repaired by homologous recombination using the sister chromatid is more likely to cause mutations, which could explain the increased divergence in sequence composition.

As more T2T genomes become available, both for more individuals in a given species and for more diverse species, future studies will be able to further reveal the contribution of STRs in driving eukaryotic evolution and adaptation. To conclude, this study analyzed the distribution and topography of STRs across 117,861 genomes from various organisms, finding that STRs are mostly enriched in eukaryotes, with viruses infecting eukaryotic hosts also showing higher STR densities. The highest STR densities were observed in the Protista kingdom and Euglenozoa phylum, while bacterial and archaeal genomes showed low STR densities. Simulation experiments showed a relative depletion of STRs from bacterial and archaeal genomes.

Despite the large number of complete genomes analyzed, our results were constrained by the limited availability of such genomes. For instance, our analyses included only four distinct protozoan phyla, and some phyla contained a disproportionate number of species compared to others. As sequencing technologies continue to advance and more complete genomes become available, further studies will be needed to elucidate the evolutionary trajectories of STRs both across and within each species.

## Materials and methods

### Data retrieval and parsing

Complete genomes were downloaded from the GenBank and RefSeq databases [69, 70] using the Genome Updater bash utility https://github.com/pirovc/genome_updater, with the following command:

./genome_updater.sh -d “refseq, genbank” -g “archaea, bacteria, fungi, plant, protozoa, vertebrate_mammalian, vertebrate_other, invertebrate, viral” -l “complete genome” -o “assembly_accessions” -f “genomic.fna.gz, genomic.gff.gz” -t 12 -m.

Assembly accessions corresponding to the same accession ID were deduplicated by prioritizing RefSeq entries over GenBank entries. This process yielded a total of 117,861 complete organismal genomes across the three domains of life and viruses, which were subsequently analyzed and integrated into the database. For each genome, the associated RNA and coding region files, as well as the GFF gene annotation file, were downloaded.

 As a separate part of our analysis, we also downloaded the T2T genomes for the following primates via the T2T-Primates consortium [71, 72]: *Gorilla gorilla*, *Pan paniscus*, *Pan troglodytes*, *Pongo abelii*, *Pongo pygmaeus*, and*Symphalangus syndactylus*, which are available at https://github.com/marbl/Primates. For our analysis, we used the linear reference genomes, which have been constructed using the diploid phased assemblies, by selecting the highest quality haplotype [71, 72]. Associated files, including genome annotations, were manually retrieved from GenomeArk hosted on AWS cluster (https://github.com/marbl/t2t-browser). These assemblies were processed and analyzed separately in Figures 7 and 8.

### Identification of STRs

We defined STRs as any genomic sequence between one and nine base pairs that repeats at least three times and is at least 10 bases long. STRs were detected using non b-gfa and RPTRF, a perfect tandem repeat finder tool [73]. We used both non b-gfa and RPTRF to systematically extract the raw perfect STRs from each complete genome available in the NCBI GenBank and RefSeq databases. For RPTRF, we used parameters maximum motif size, M = 50,000, and minimum length, t = 1.

For non b-gfa we used the following command with default parameters:

non-B_gfa/gfa -seq $GENOME -out $DEST -skipAPR -skipSlipped -skipCruciform -skipTriplex -skipWGET -skipIR -skipMR -skipDR -skipZ -skipGQ.

The pipeline was split across multiple nodes on the High-performance computing system, each node processing a distinct set of complete genomes. The C + + script processed each chromosome in parallel, passing the derived sequences into a custom Python script. The Python script was created to validate, parse, and store all STRs and derive the STRs by bounding the consensus sequence length between one and nine base pairs. Additionally, we filtered any motif less than 10 bases long and repeating less than three times. Furthermore, consensus sequences containing N or other characters not originating from the nucleotide alphabet {A, G, C, T} were filtered out from the extracted STR dataset. Finally, another custom Python script was integrated into the existing pipeline to extract the perfect STRs of mononucleotide repeats, and then concatenated the two outputs into a single dataset, which was saved in a parquet-snappy compressed format.

### Calculation of average STR density in each taxonomic rank

In our database, we represent each species as a set of individual NCBI assembly accessions$\left\{A_1,A_2,...A_{n\left(S\right)}\right\}$, where $n\left(S\right)$ represents the total number of distinct complete genomes that are of type $S$. For each taxonomic rank $R$, containing a sequence of species$\left\{S_1,S_2,...,S_{m\left(R\right)}\right\}$, where $m\left(R\right)$ denotes the total number of distinct species of rank $R$, we calculate the STR density per kB, denoted as $F\left(R\right)$, as the average of STR densities across all the species of rank$R$, i.e.:

$$F\left(R\right)=10^3\frac{\sum_{j=1}^{m\left(R\right)}F\left(S_j\right)}{m\left(R\right)}$$

where $F\left(S_j\right)$ represents the individual STR density on the species levels, defined similarly as the average of STR density across all the assembly accessions corresponding to species $S_j$.

### Identification of STRs in simulated organismal genomes

To estimate the expected abundance of STRs, we used the uShuffle package (Jiang et al. 2008) [50]. For each genome, we employed the Shuffle class from the ushuffle Python package to generate a dinucleotide-preserving shuffled version of the genome. Genomes were processed in 5-million-base chunks, each of which was shuffled independently to retain its original dinucleotide composition. The resulting simulated genome thus consisted of multiple such shuffled chunks. We applied this procedure to all available chromosomes of each genome, producing a dinucleotide-preserving control genome per organism. STRs were then extracted from the simulated genomes using the same method as for the original sequences. Finally, we compared STR densities (per kilobase) between the shuffled and original genomes, quantifying enrichment as the ratio of observed to expected STR bps using the following formula:

$$FE\left(O,E;G\right)\;=\frac{O-E}{O+E}$$

where$FE\left(O,E;G\right)$ is the enrichment,$O$ is the observed, and $E$ is the expected STR occurrences in a given organismal genome $G$. Given the definition above, the enrichment function $FE\left(O,E;G\right)$ takes values in the interval$\left[-1,1\right]$, with the extreme values − 1 indicating maximal depletion and 1 maximal enrichment, respectively. If the genome initially contained no STRs, and its corresponding shuffled genome was also empty, it was natural to assume that there is neither depletion nor enrichment, and thus, in such cases where$O=E=0$, we define $FE\left(O,E;G\right)=0$. The statistical significance of the enrichment between the organismal and the simulated, dinucleotide-preserving shuffled genomes was assessed using a two-tailed independent t-test, adjusting for multiple comparisons using Benjamini-Hochberg procedure.

### Derivation of the tree of life

We used the NCBI taxdump database to retrieve the full taxonomic lineage for each genome assembly accession. This was done using the ncbitax2lin Python package, which maps species-level taxonomic identifiers (taxIDs) to their complete lineage, spanning all ranks from family to domain. To ensure comprehensive taxonomic coverage, we also incorporated metadata from the NCBI assembly_summary.txt file, which provides detailed lineage information for each genome in our dataset. Since some groups—such as protists—are not explicitly annotated; we used the *group* column in the assembly summary to assign unclassified entries to higher taxonomic categories. For example, assemblies listed under the “protozoa” group were assigned to the Protista kingdom. Similarly, we used the *group* column to fill in missing lineage information on the kingdom and domain levels; for instance, genomes assigned to the “fungi” group were mapped to the Fungi kingdom and the Eukaryota domain, genomes assigned to the “plant” group were mapped to the Plantae kingdom and the Eukaryota domain, and, similarly, “vertebrate mammalian” group were mapped to the Metazoa kingdom and the Eukaryota domain.

### Identification of viral host and molecular type

To map each viral organismal genome to a particular host we downloaded NCBI viral metadata from genome reports:

https://ftp.ncbi.nlm.nih.gov/genomes/GENOME_REPORTS/viruses.txt.

Eukaryotic host viruses were defined using the host column from the genome reports dataset. In particular, we identified eukaryotic hosts of any viruses annotated as plants, eukaryotic algae, invertebrates, fungi, land plants, human, protozoa, vertebrates. Bacterial and archaeal host viruses were simply identified by the same column. Viruses with hosts across the domains were omitted from this analysis. Finally, we mapped a proportion of the viral organismal genomes in our dataset to the genome reports dataset and calculated the STR density in each host category as described in Estimation of STR density across genomes section.

### Estimation of STR density across genomes and genomic subcompartments

To accurately estimate the STR density in each organismal genome, we used the BEDtools merge command to merge the overlapping STR sequences and subsequently calculate the total length of the merged sequences for each given file. Finally, the STR density for each organismal genome was calculated by dividing the total length of the merged sequence by its genome size, multiplied by 1,000 to report values in kB. In order to mitigate bias arising from multiple assemblies corresponding to the same species, the STR density was first averaged within each individual species, before reporting the average STR density across higher taxonomic ranks, and, in particular, in phyla, kingdoms, and domains. Additionally, STR density across different genomic subcompartments was assessed by dividing the total overlap number of base pairs of STRs within each subcompartment by the overall length in base pairs of these subcompartments. Coordinates for subcompartments were sourced from the relevant NCBI GFF files, and any overlapping annotations within a subcompartment were merged into a single union using BEDTools merge command. The average STR density was also computed across species within the same taxonomic group, either throughout the entire genome or within specific genomic subcompartments. Species not associated with a GFF file were omitted from this analysis. Furthermore, certain viral species that did not contain any relevant genomic compartment, such as genes, exons, or CDS, were also skipped during the density extraction.

### Prevalence of STR motifs across taxonomic ranks

We estimated the prevalence of STR consensus motifs across different taxonomic ranks. For each repeating unit length, STR motifs were grouped into mutually exclusive classes. Each class included all motifs that could be derived from one another through cyclic shifts and reverse complement operations. The representative of each class was defined as the lexicographically minimal motif.

For each species, we calculated the proportion of a given representative motif by dividing the total sequence length of STRs matching that motif by the total length of all STR sequences found in the species’ assemblies. We then averaged these proportions across species within each domain (Fig. 3) and phylum (Fig. 4), providing an estimate of the true prevalence of each representative motif at these taxonomic levels.

### T2T chromosome STR density

Each chromosome of the T2T genomes was partitioned into mutually exclusive 100kB genomic bins of equal length. Each STR start and end coordinate was assigned to a particular bin from 1 to N, according to the following formula:

$$\mathrm{bin}\left(\mathrm x;\mathrm C\right)=1+\left[\frac{\mathrm{Nx}}{\mathrm C}\right]$$

where x is the start or end coordinate of the STR, C is the chromosome size and$\left[.\right]$ denotes the floor function. Subsequently, for each bin we used BEDtools coverage command to evaluate the total STR density, which was then divided by the genome-wide STR density to determine the fold enrichment. In cases where the start and the end of the STR were assigned a different bins, these STR bases were counted in all of the intermediate bins. Finally, for each bin we decomposed the STR bases into proportions corresponding to the various repeating unit lengths of the STR consensus sequence.

### Estimation of PWM across genomes

The positional weight matrix (PWM) for STR motifs was extracted at a generated window of 500 bp upstream and downstream from TSSs or TESs. The process involved a custom Python script which utilized BEDTools to extract the intersections of tandem repeats with genic compartments derived from GFF files. At each intersection, the tandem repeat sequence was saved as a separate column, along with the corresponding gene strand location. Finally, the script constructed a numpy vector array (1, 1001) which stored the occurrences for each nucleotide of tandem repeats relative to the TSSs and TESs coordinates at a particular position, ensuring the motif is translated to the same strand as to where the genic region lies. This process was repeated for repeating unit lengths 1 to 9, for all the available organismal genomes in the NCBI database associated with a GFF file. For the creation of logoplots we used the Logomaker Python package in combination with Matplotlib [74]. The (4, 1001)-matrix containing the probability of a nucleotide occurring at each position in the expanded window, was calculated by using the Bayes estimator:

$$\mathrm E\;\left[{\mathrm\theta}_{\mathrm i}\vert\alpha\right]=\frac{{\mathrm\theta}_{\mathrm i}+{\mathrm\alpha}_{\mathrm i}}{\sum_{\mathrm i=1}^4\left({\mathrm\theta}_{\mathrm i}+{\mathrm\alpha}_{\mathrm i}\right)}$$

of a Dirichlet prior, where, $a=\left(\alpha_1,\alpha_2,\alpha_3,\alpha_4\right)$ is the pseudocount vector, and $\theta=\left(\theta_1,\theta_2,\theta_3,\theta_4\right)$ is the probability vector of nucleotide from the nucleotide alphabet $\:\varPi\:=\{A,\:C,\:T,\:G\}$ occurring at a particular position. In our analysis, we used the Dirichlet prior as the prior distribution for the probability of each nucleotide occurring at each position.

### Estimation of STR density relative to TSSs and TESs

To examine the distribution of short tandem repeats (STRs) near annotated CDS and mRNA boundaries, we used the “gene” features from NCBI GFF files to characterize STR occurrence relative to prokaryotic translation start and end sites, as well as eukaryotic transcription start sites and 3′-cleavage sites. Genomes lacking gene-level annotations in their GFF files were excluded from this analysis. For each set of coordinates, we created 500 bp windows centered around these genic landmarks using annotations from NCBI GFF files. Within each window, we quantified the number of STR base pairs at each position for each organismal genome, analyzing each STR repeat unit separately. Relative enrichment at each position was calculated as the number of STR base occurrences divided by the mean number of occurrences across the entire window. To address potential biases due to uneven representation of species and families across domains, we constructed two separate 95% confidence intervals using Monte Carlo resampling with replacement (*N* = 1,000). The inner confidence interval was derived by bootstrapping at the species level, and the outer interval at the family level. This approach yielded a normalized measure of the conditional probability of observing an STR base, given that an STR is present, at each position relative to TSSs and TESs across diverse taxonomic groups.

#### Mutation calling from bacterial complete genomes

We downloaded all available complete genomes of 87 bacterial species from the NCBI RefSeq and GenBank databases. For each species, we selected a reference genome directly from the combined assembly summary of both databases. Whole genome pairwise alignments were then performed using minimap2 [75] against all available strains of the same species. Each generated BAM file was sorted with samtools sort, and polymorphisms were called using bcftools mpileup and bcftools call with ploidy set to 1 [76]. The resulting variants were normalized using bcftools norm to left-align and standardize calls against the reference genome. High-quality variants were retained by filtering with bcftools filter, using a minimum Phred-scaled quality score of 60, and a minimum mapping quality of 50 at each polymorphic locus. After filtering, polymorphic loci were classified into three mutually exclusive categories: substitutions, small deletions (≤ 50 bp), and small insertions (≤ 50 bp). Variants larger than 50 bp were not analyzed.

 To visualize the relationship between STR density and polymorphic loci, we used the Seaborn library. Spearman correlation and linear regression were performed using the SciPy linregress function, with p-values adjusted for multiple comparisons via the Benjamini-Hochberg procedure. Outlier influence in the regression was assessed using Cook’s distance from the statsmodels Python module. The overlap between STRs and polymorphic loci was determined using bedtools coverage [77]. To assess the statistical significance of observed overlaps, we generated control regions complementary to STRs using bedtools shuffle, preserving the original STR length distribution. To account for sequence composition bias between genic and intergenic regions, separate control groups were created for genic and intergenic STRs: STRs overlapping ≥30% of a coding region were classified as genic. The proportion analysis of polymorphisms overlapping STRs was then repeated for these stratified controls. For each bacterial species, we calculated the observed ratio of polymorphic sites overlapping at least one STR to the total number of polymorphic sites. The same ratio was computed for the matched control regions, and fold enrichment was calculated as the ratio of the observed proportion to the control proportion. A pseudocount of 1 was added to the denominator to account for cases where no control regions overlapped with polymorphic loci. This analysis was performed separately for each mutational type across all bacterial species with non-zero STR density. Statistical significance was assessed using a one-tailed Fisher’s exact test, with p-values adjusted for multiple comparisons using the Benjamini-Hochberg procedure.

## Supplementary Information

Supplementary Material 1.

## Data Availability

All the source data and the scripts in this study can be found in Zenodo repository (https://zenodo.org/records/17692318) [78]. The complete organismal genomes and their annotations were downloaded from NCBI [77]. Primary haplotypes of the Great Ape T2T genomes for the following primates: Gorilla gorilla, Pan paniscus, Pan troglodytes, Pongo abelii, Pongo pygmaeus, and Symphalangus syndactylus were retrieved via the T2T-Primates consortium [58, 71]. Associated files, including genome annotations, were manually retrieved from GenomeArk hosted on AWS cluster (https://github.com/marbl/t2t-browser).All code is released under the MIT License.

## Code availability

The GitHub code and all the related material is provided at: https://github.com/Georgakopoulos-Soares-lab/treeoflifeSTR.
