# Supplementary material for: The repertoire of short tandem repeats across the tree of life
Source: Genome Biol. 2025 Dec 12;26:425. doi: 10.1186/s13059-025-03893-z (PMC12699852; doi:10.1186/s13059-025-03893-z)
Supplement: Supplementary file 1 — Supplementary Material 1. [file 13059_2025_3893_MOESM1_ESM.docx]

**Supplementary Material**

**
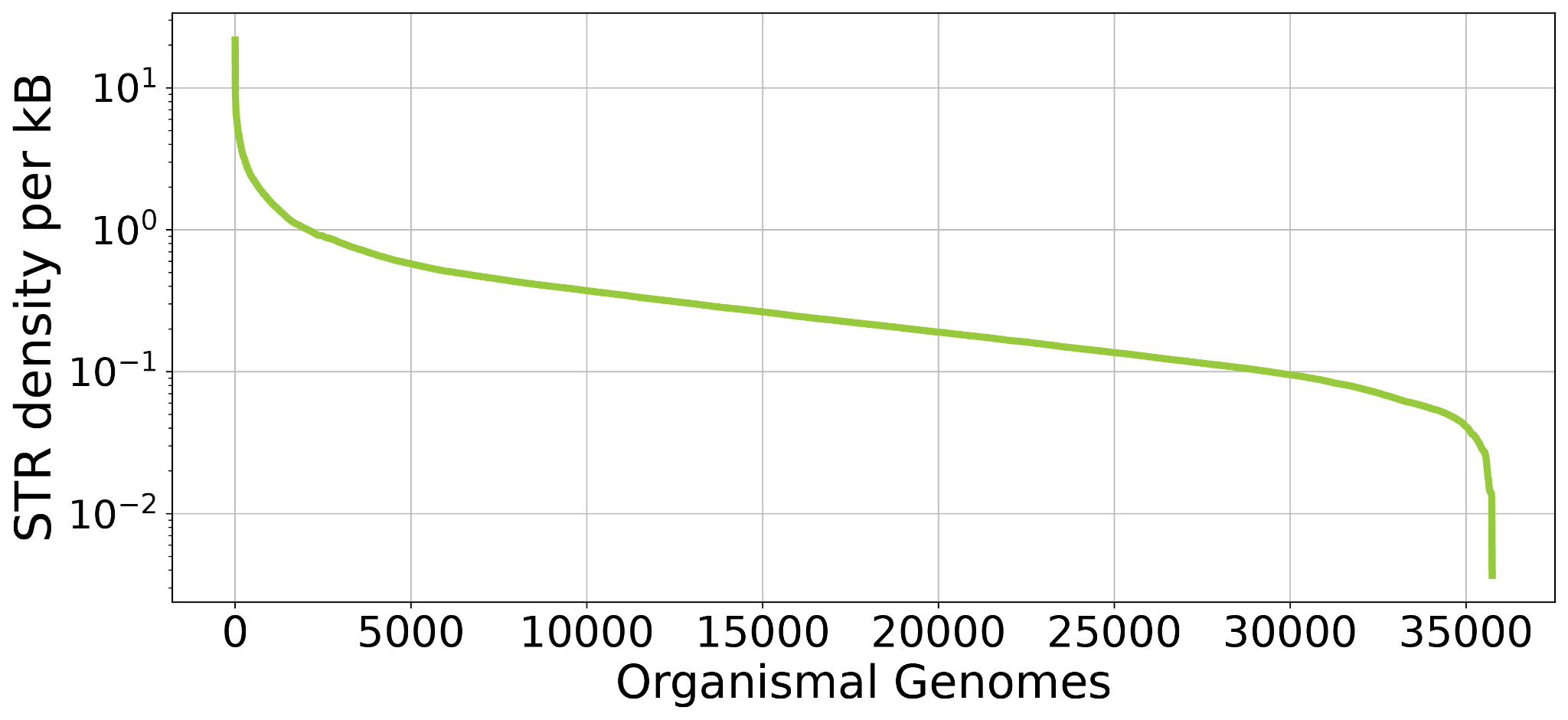
**

**Fig S1: STR density per Kb per organismal genome averaged on the species level.**

**
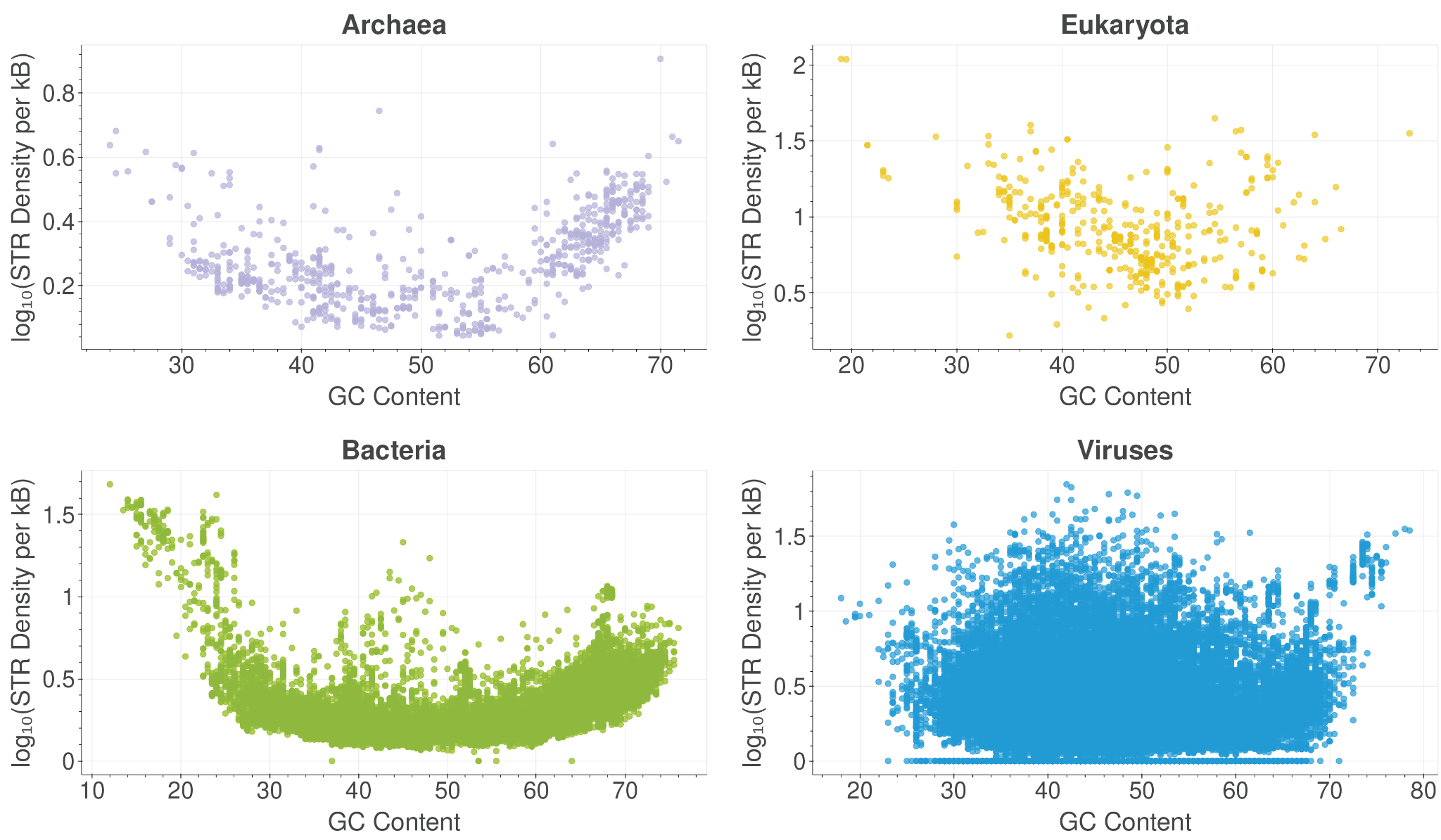
**

**Fig S2: STR density per kB vs GC content per organismal genome across the three domains of life and viruses.**

**A**

**
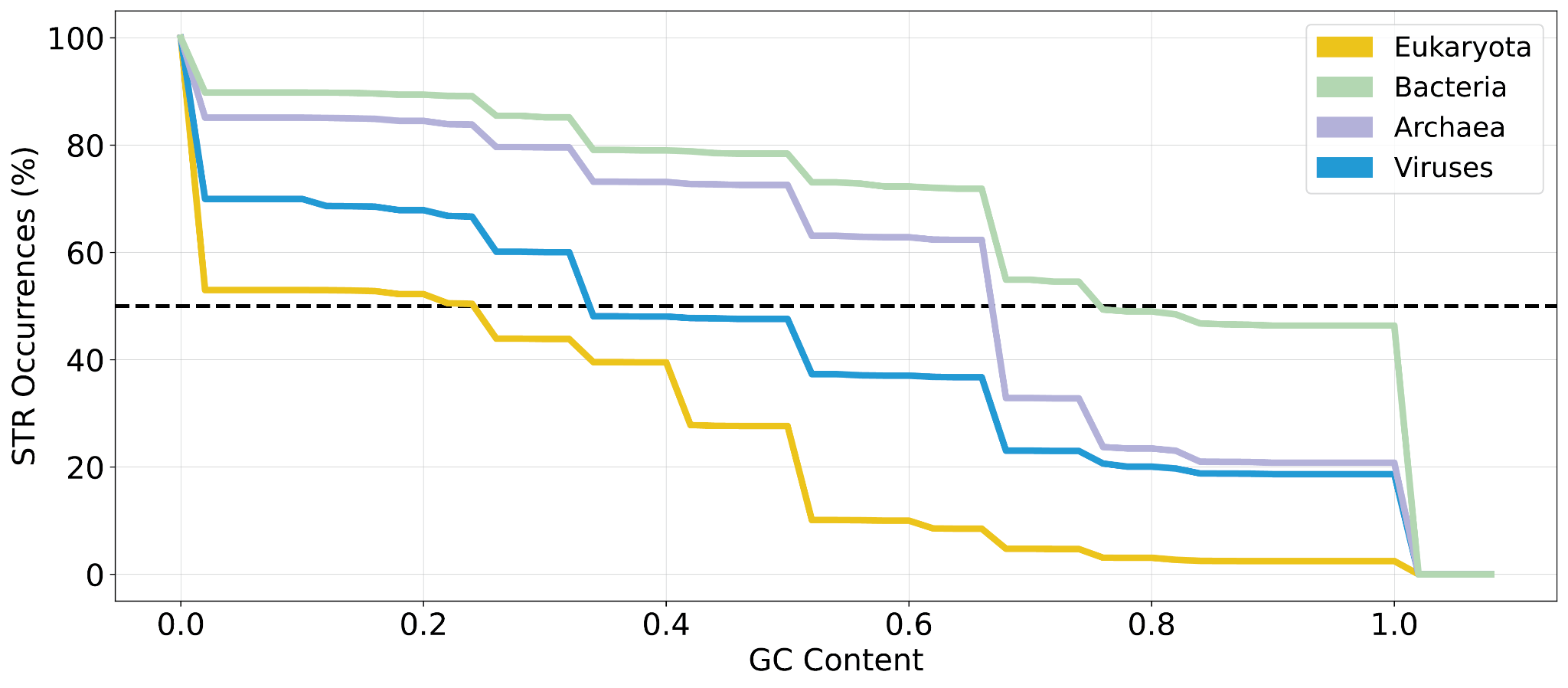
**

**B**

**
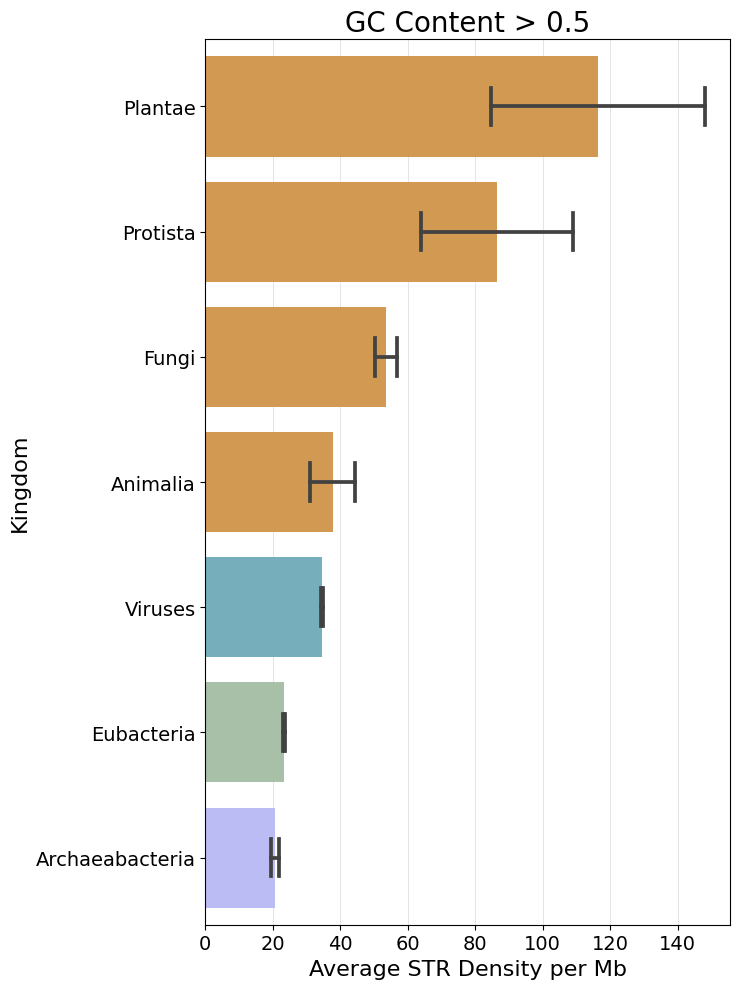
**

**Fig S3: STR occurrence density for various GC Content thresholds.** Results shown **A.** for the three domains of life and viruses and **B.** for kingdoms. Coloring in B. is done based on the domain of life.

**
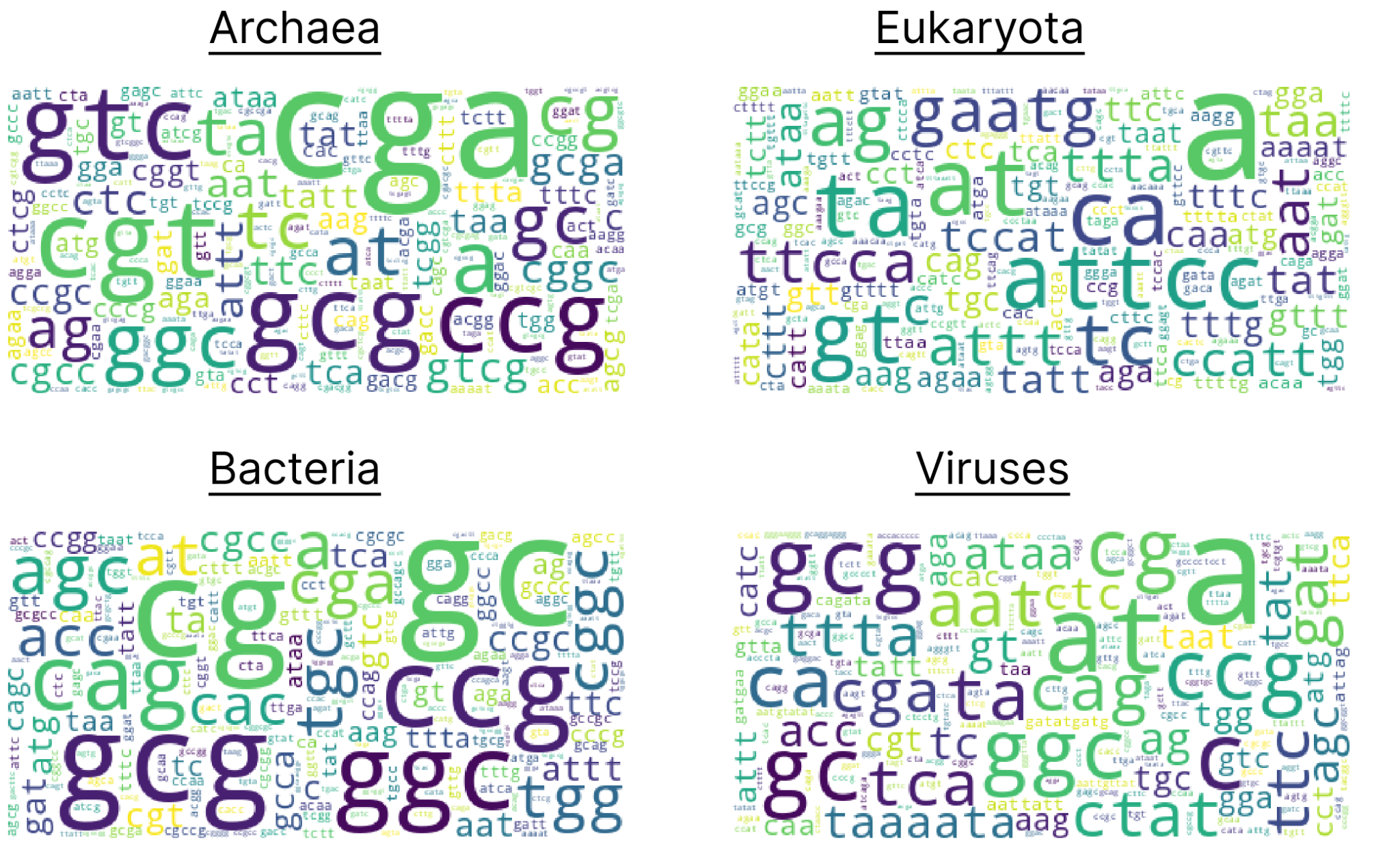
**

**Fig S4: Frequency of most frequent STR motifs across the three domains of life and viruses.**

**
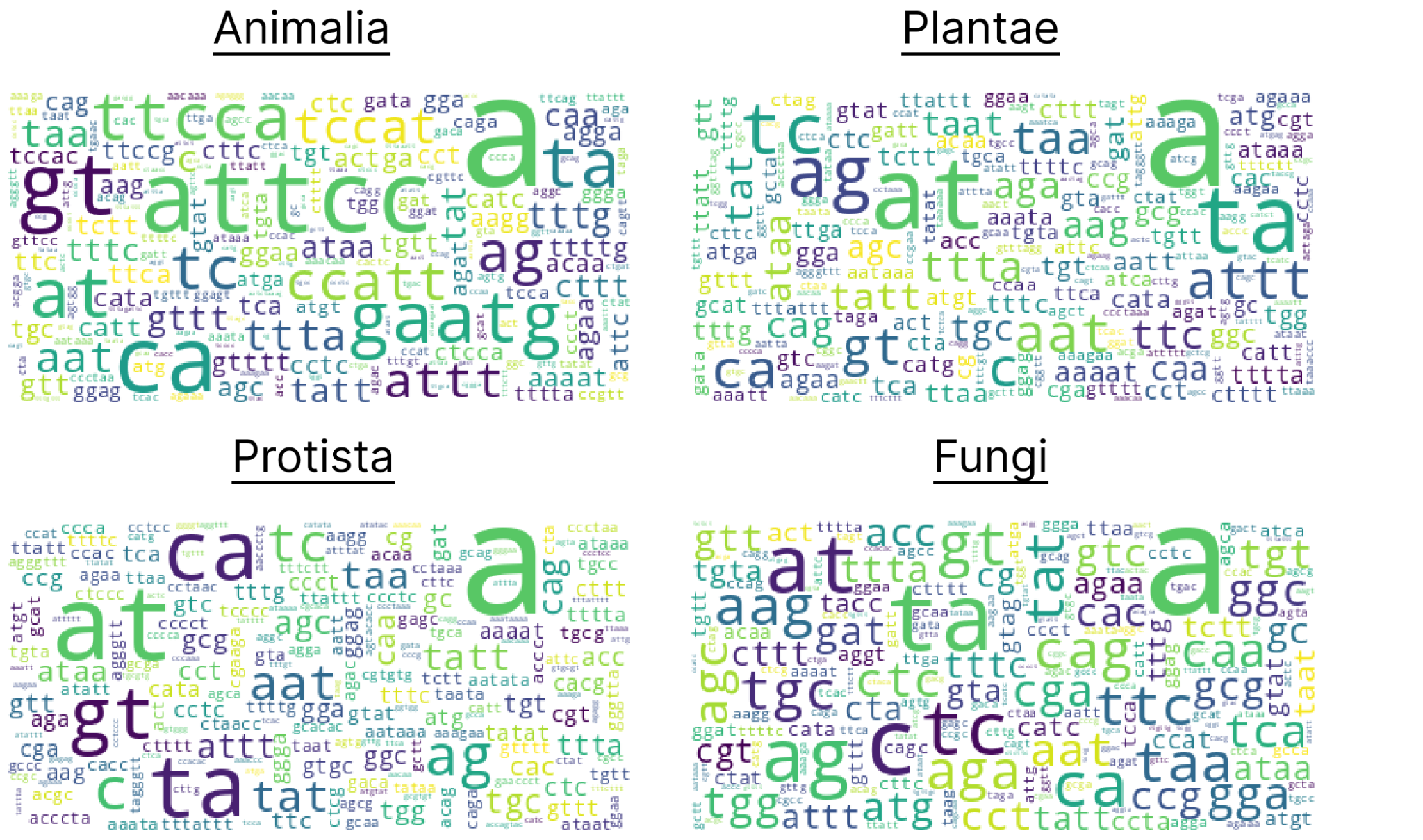
**

**Fig S5: Frequency of most frequent STR motifs across eukaryotic kingdoms.**

**
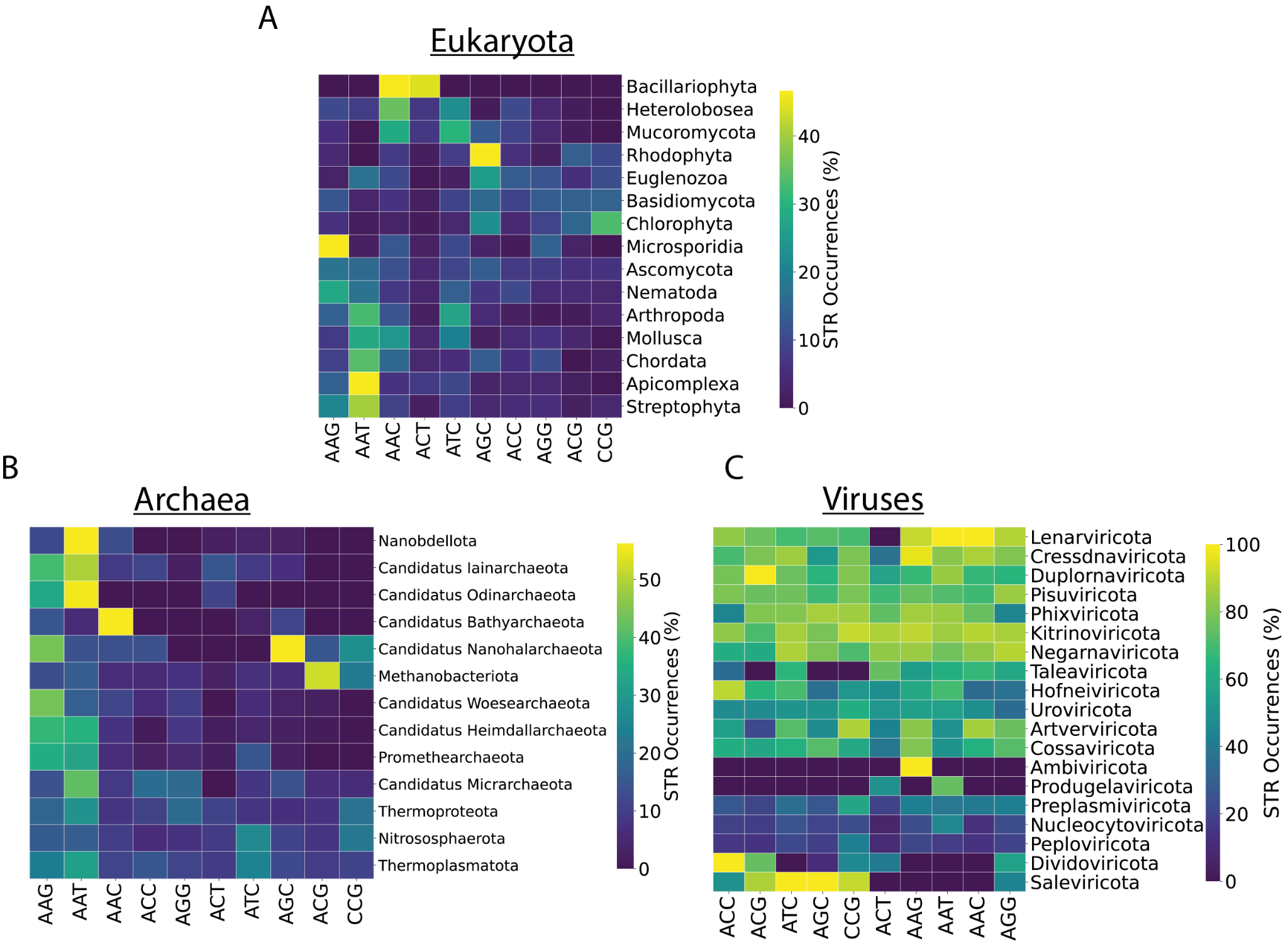
**

**Fig S6: Hierarchical clustering of the prevalence of trinucleotide STRs across A. eukaryotic, B. archaeal and C. viral phyla.** The values represent the empirical mean of the proportion of each trinucleotide repeat across the species for each phylum. The proportions were calculated on the species level from all the individual organismal genomes.

**
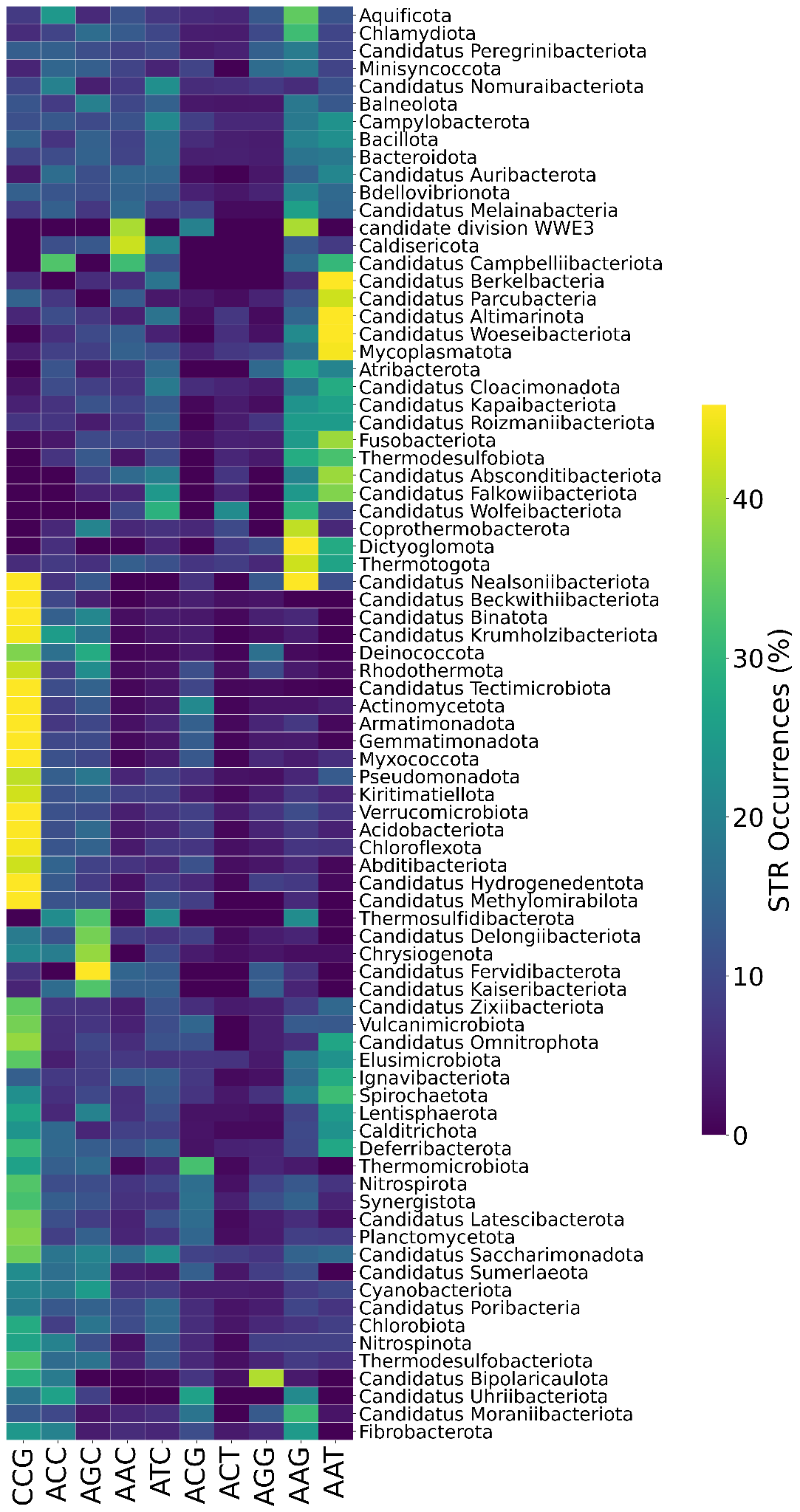
**

**Fig S7: Hierarchical clustering of the prevalence of trinucleotide STRs across bacterial phyla.** The values represent the empirical mean of the proportion of each trinucleotide repeat across the species for each phylum. The proportions were calculated on the species level from all the individual organismal genomes.

**
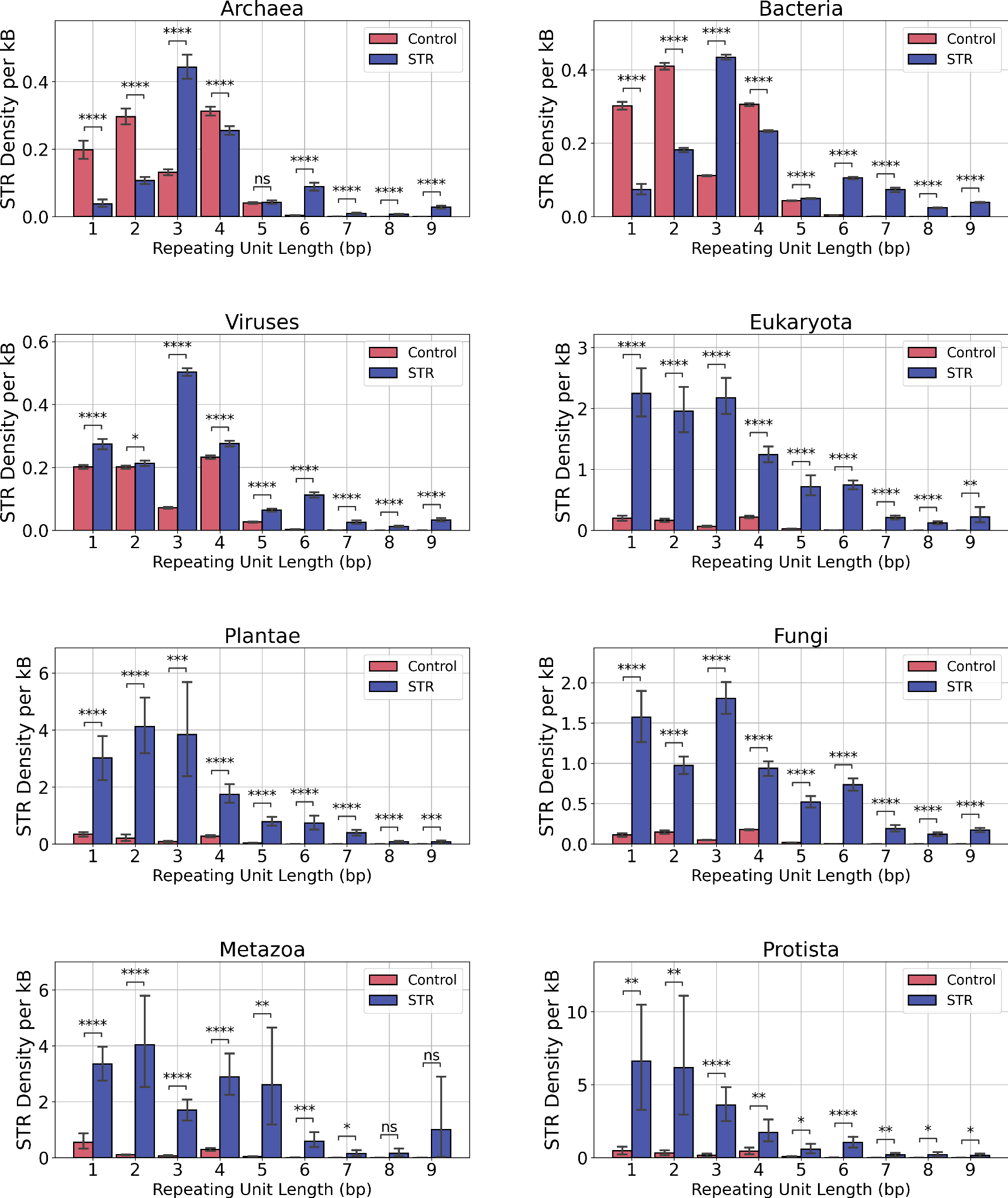
**

**Fig S8: Observed versus expected STR density across different taxa.** Results are shown for repeat unit lengths ranging from one to nine base pairs. Blue indicates STR density in real genomes, while purple represents STR density in simulated genomes. Pairwise comparisons were performed using two-tailed independent t-tests, with p-values adjusted for multiple comparisons using the Benjamini–Hochberg method. Asterisks denote significance levels: p < 0.05 (*), p < 0.01 (**), p < 0.001 (***), and p < 0.0001 (****).

**
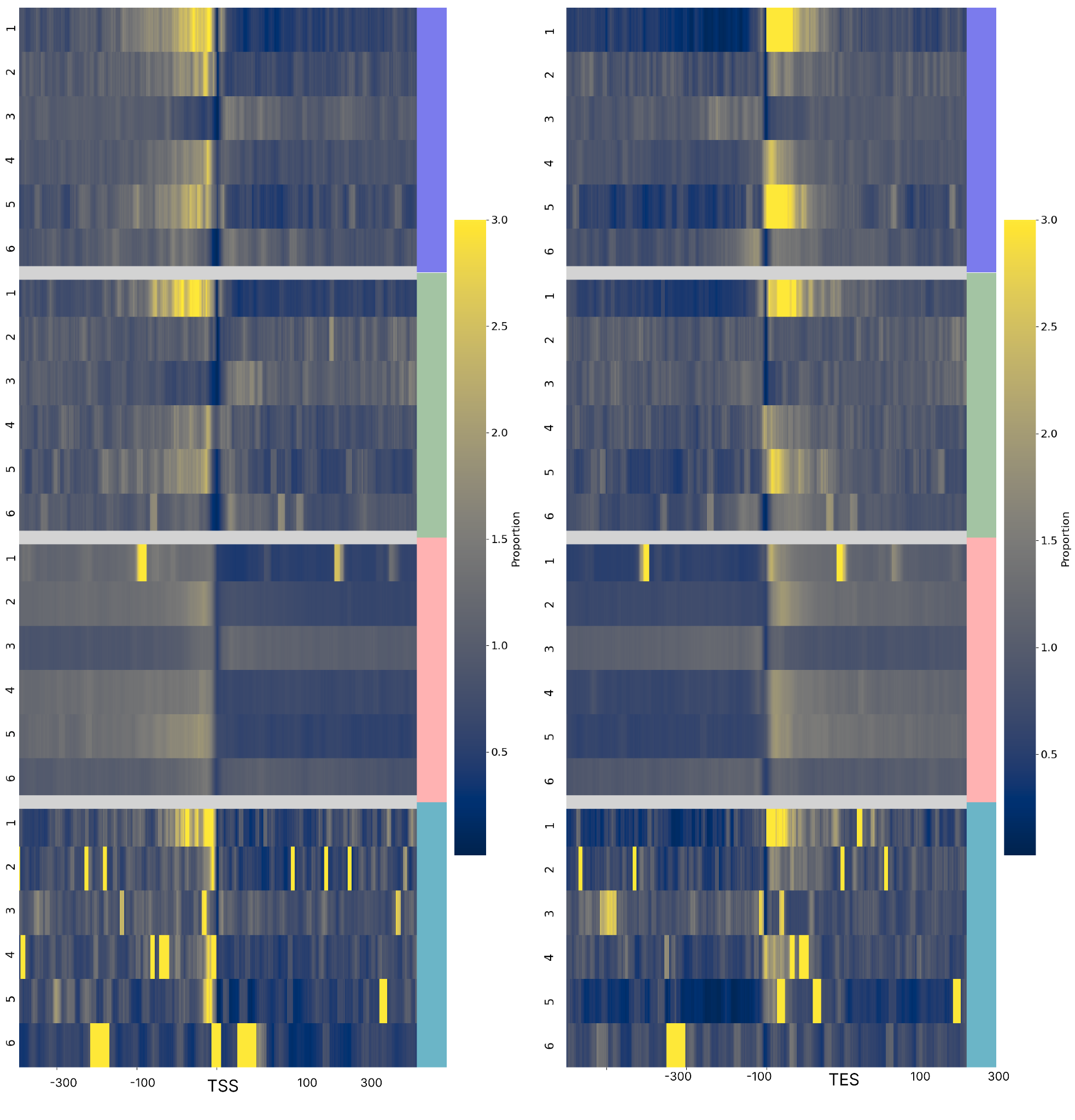
**

**
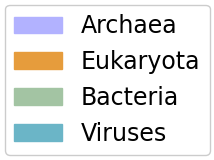
**

**Fig S9: Distribution of STRs relative to TSSs/TESs in the three domains of life and viruses.**

**
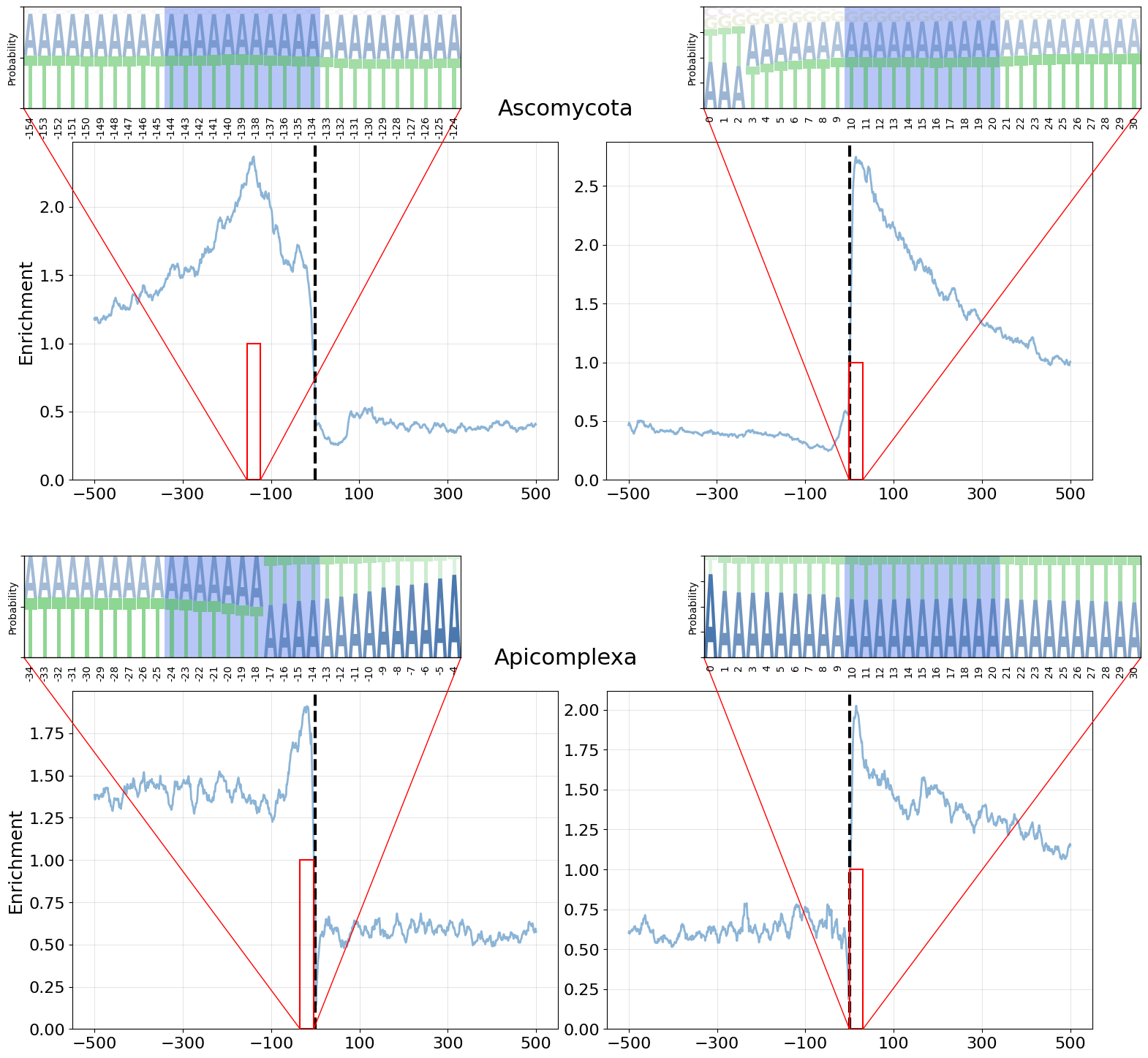
**

**
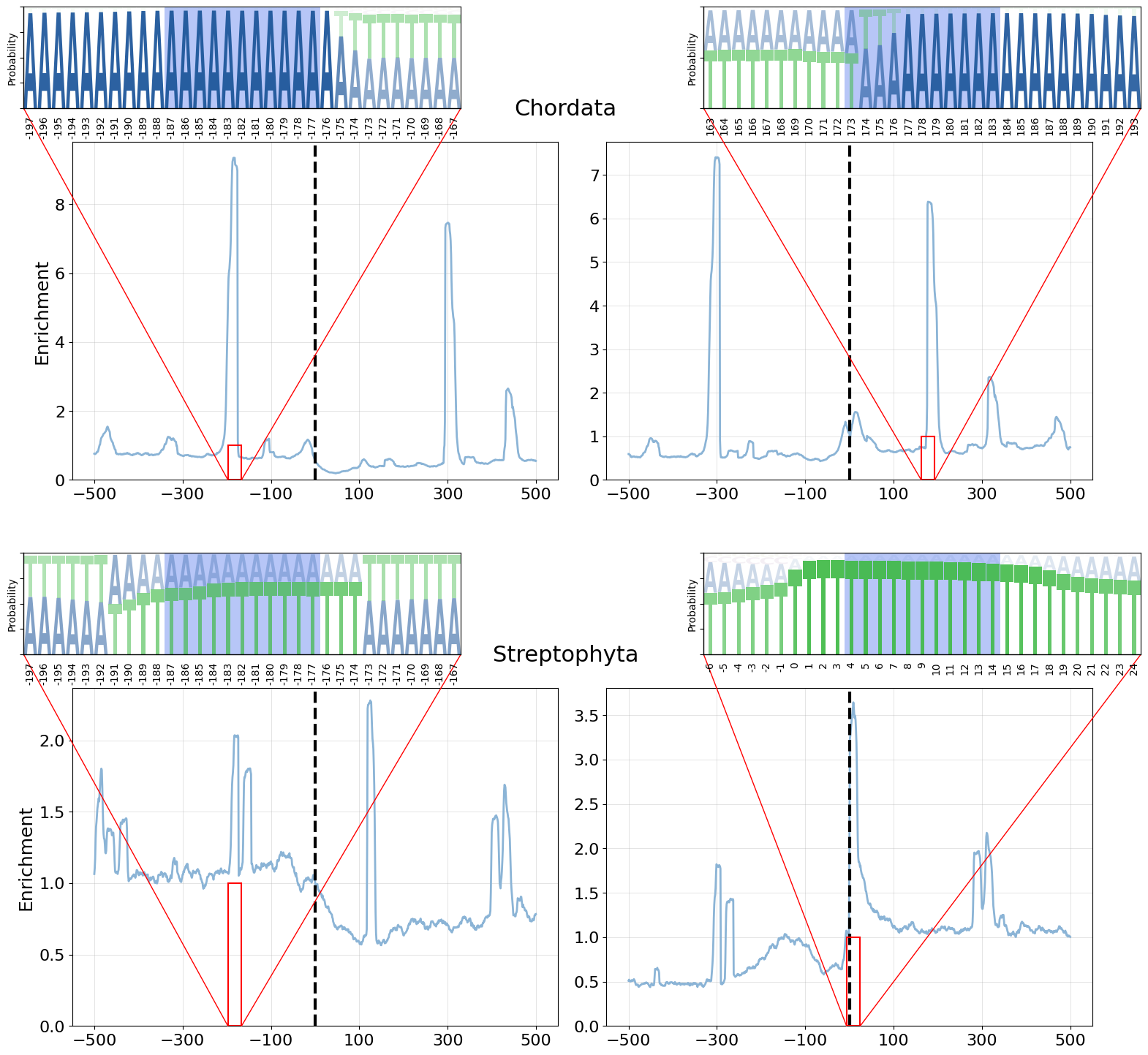
**

**Fig S10: Distribution of mononucleotide STRs in Ascomycota, Apicomplexa and Streptophyta.**

**
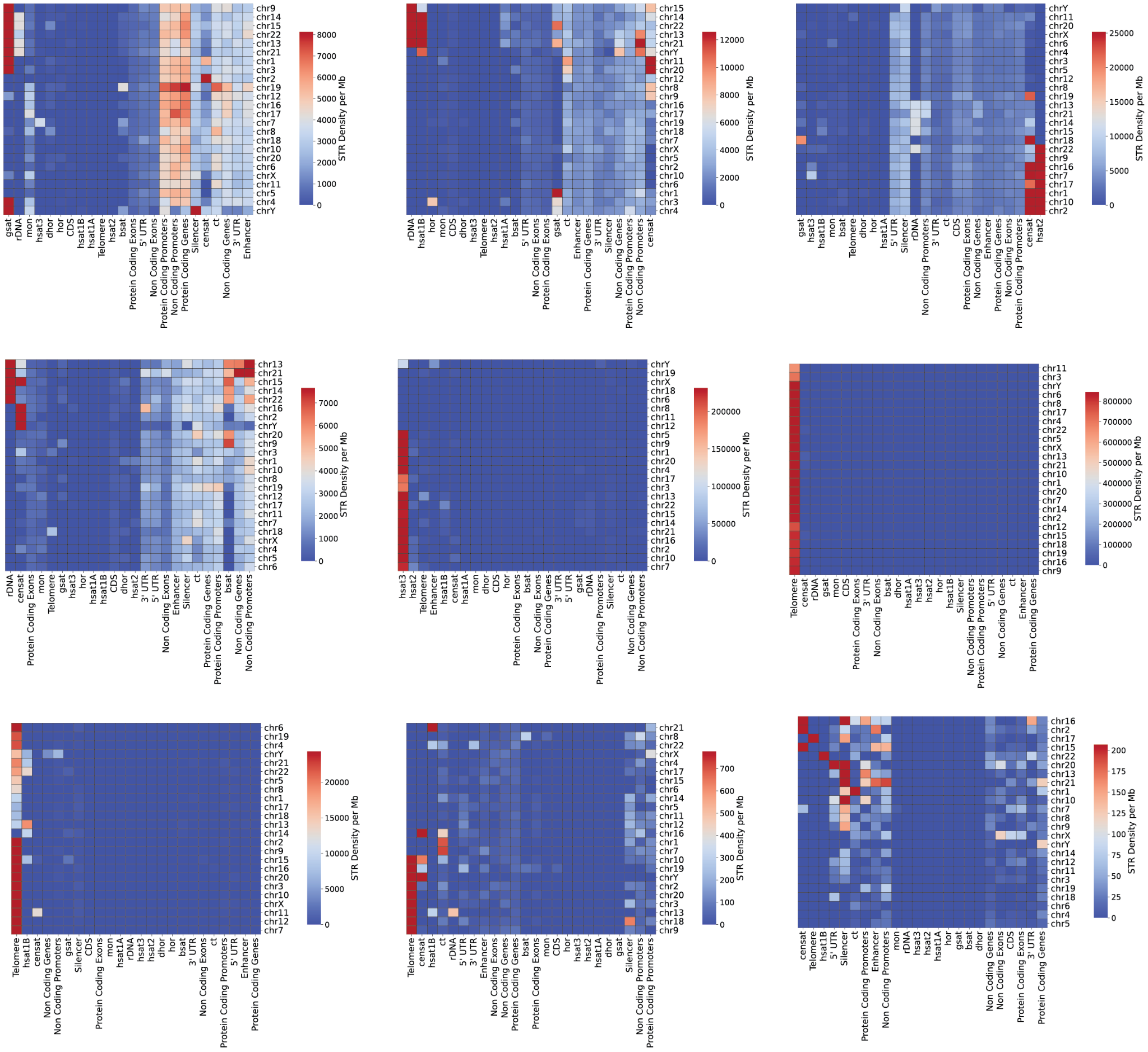
**

**Fig S11: STR Density across the human genome sub-compartments separated by STR repeat length.** Repeats include inactive αSat HOR (hor), divergent αSat HOR (dhor), monomeric αSat (mon), classical human satellite 1A (hsat1A), classical human satellite 1B (hsat1B), classical human satellite 2 (hsat2), classical human satellite 3 (hsat3), beta satellite (bsat), gamma satellite (gsat), other centromeric satellites (censat) and centromeric transition regions (ct). Results shown sequentially for one to nine bp STR units.

**
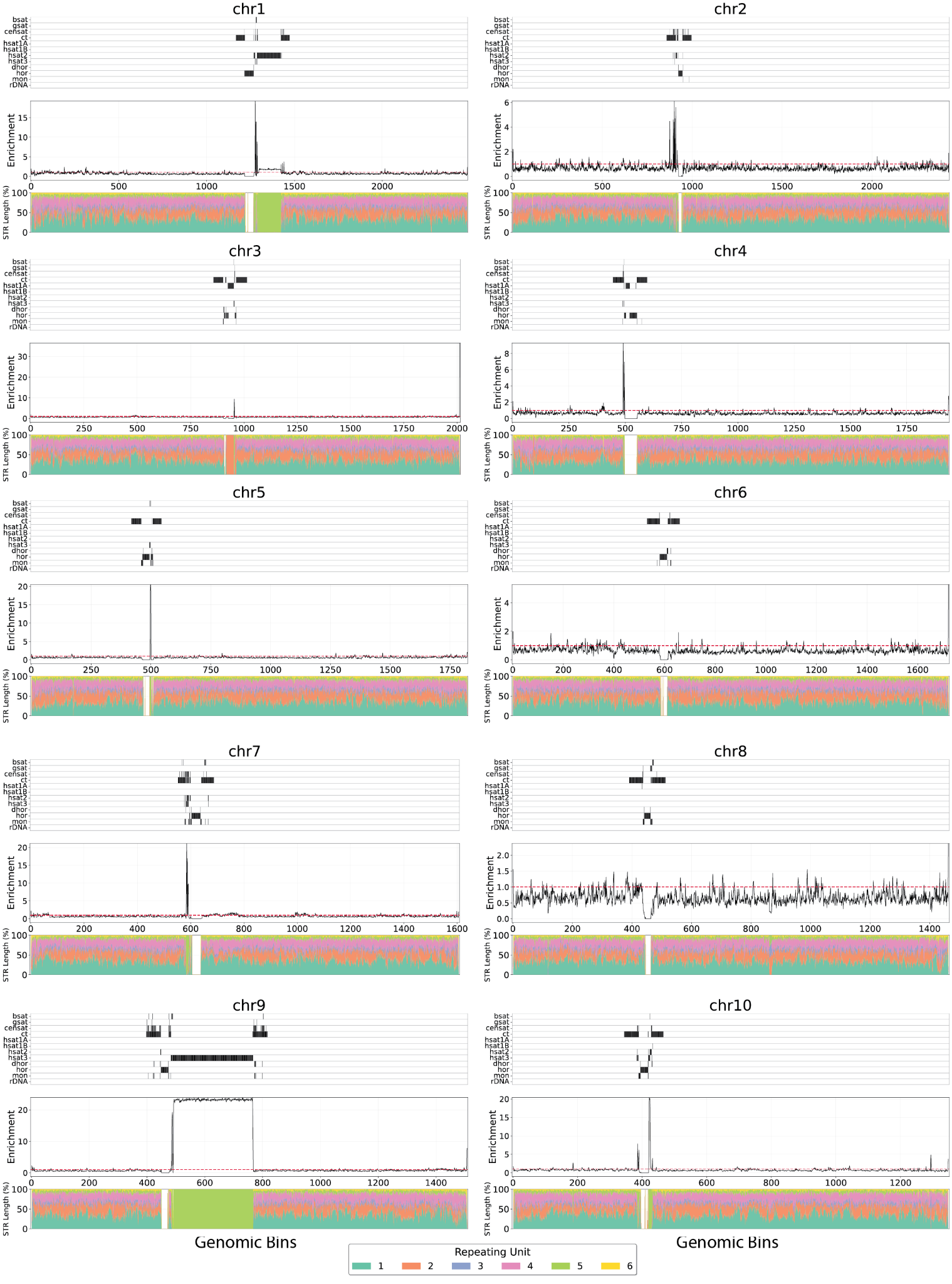
**

**Fig S12**: **Characterization of STRs across chromosomes in the T2T reference human genome, each one equipartitioned into mutually exclusive regions of 100kb length.** Schematics show the distribution of STRs across different human chromosomes. The top panel aligns the position of centromeric and pericentromeric regions. The heatmap shows the different types of pericentromeric and centromeric repeats, with black color representing the presence of the repeat in that genomic region. Line plots show the STR fold enrichment at each genomic 100kb length bin for a chromosome. Stacked barplots show the results for different STR repeating unit lengths. Repeats include inactive αSat HOR (hor), divergent αSat HOR (dhor), monomeric αSat (mon), classical human satellite 1A (hsat1A), classical human satellite 1B (hsat1B), classical human satellite 2 (hsat2), classical human satellite 3 (hsat3), beta satellite (bsat), gamma satellite (gsat), other centromeric satellites (censat) and centromeric transition regions (ct).

**
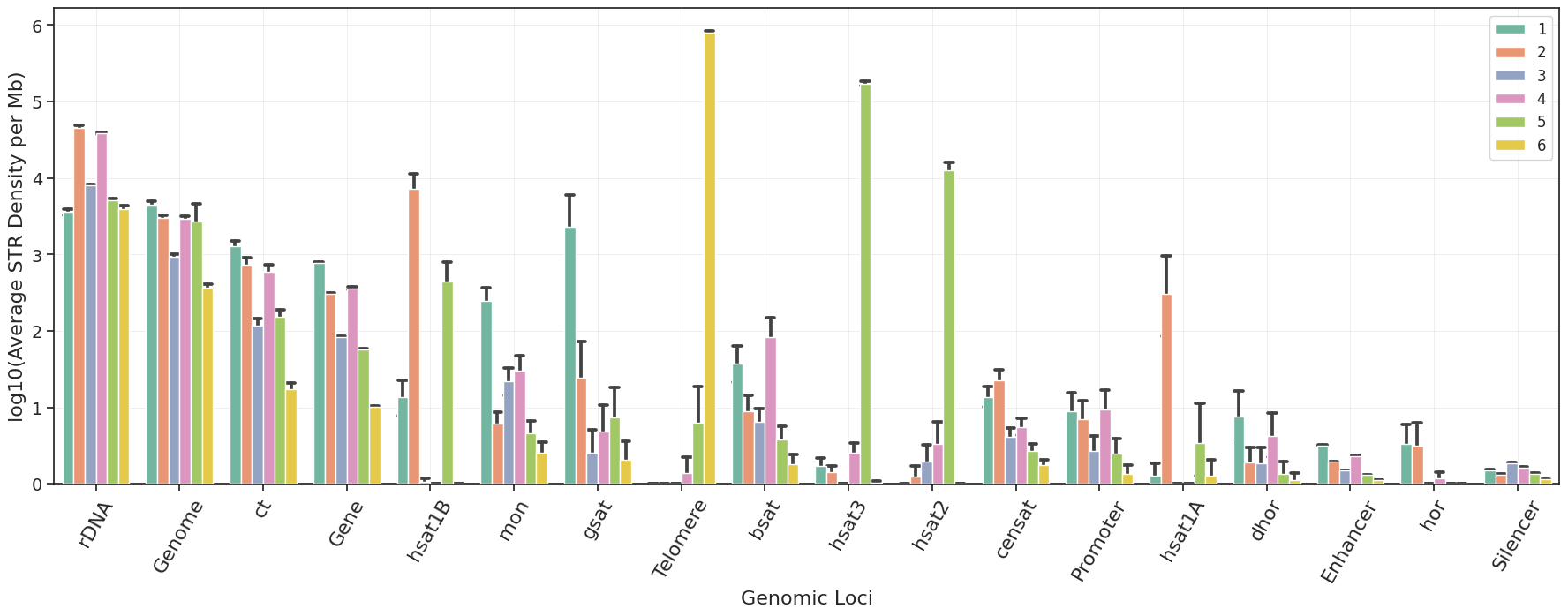
**

**Fig S13: STR Density across human genome sub-compartments including centromeric repeats, separated by STR repeat length.** Repeats include inactive αSat HOR (hor), divergent αSat HOR (dhor), monomeric αSat (mon), classical human satellite 1A (hsat1A), classical human satellite 1B (hsat1B), classical human satellite 2 (hsat2), classical human satellite 3 (hsat3), beta satellite (bsat), gamma satellite (gsat), other centromeric satellites (censat) and centromeric transition regions (ct).

**
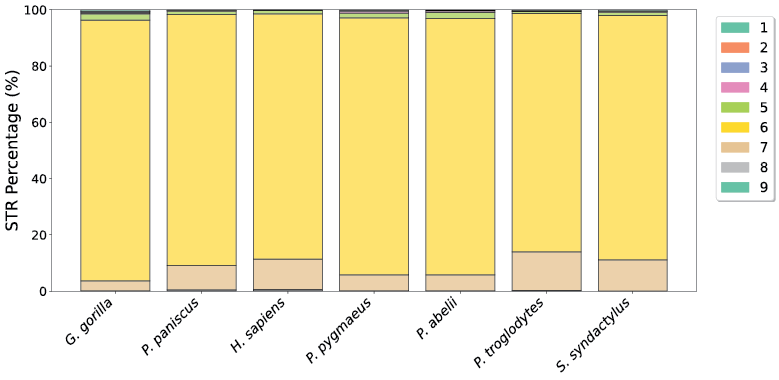
**

**Fig S14: STR composition of telomeres across six primate species.**


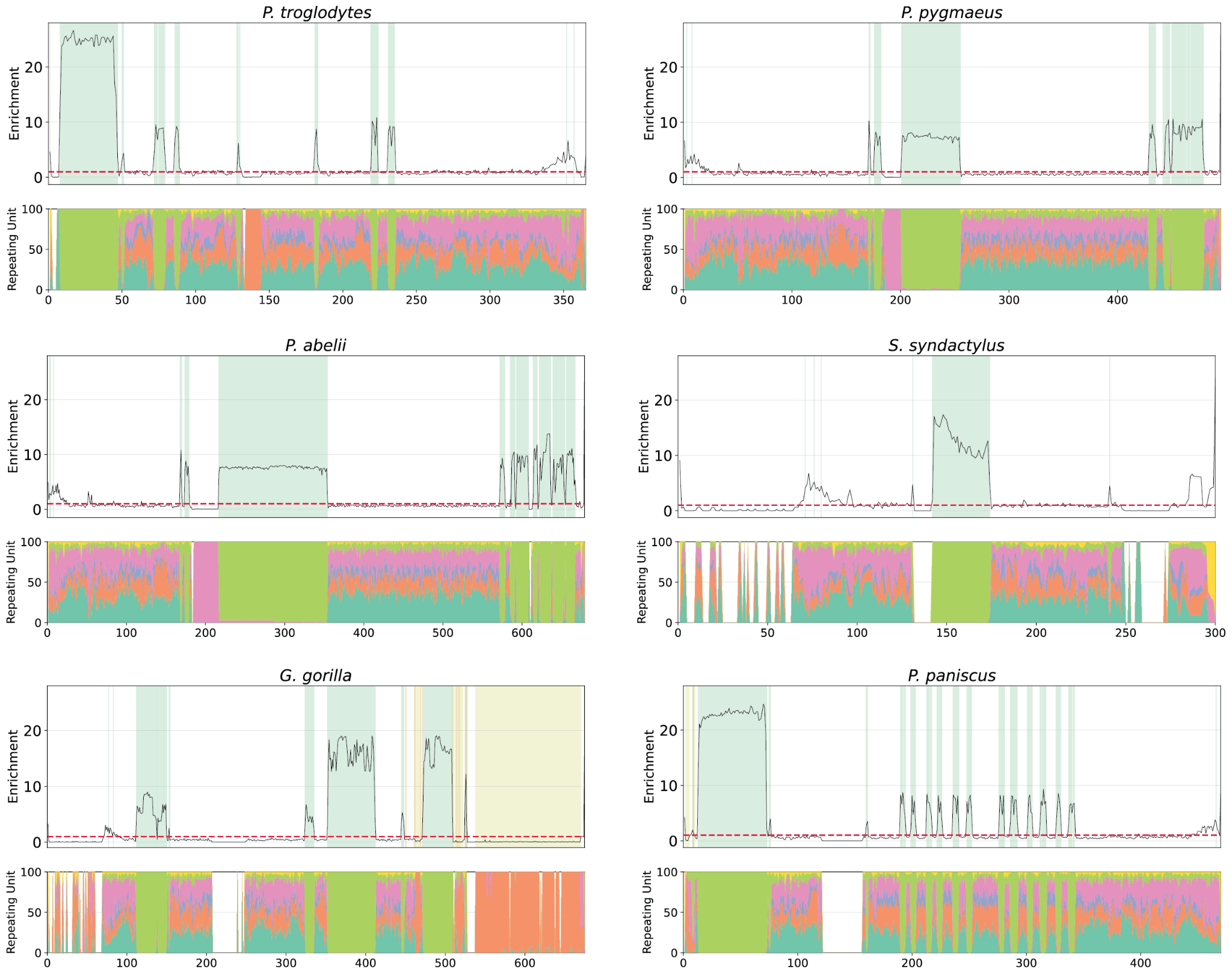


**Fig S15: Characterization of STRs across the Y chromosomes of the T2T primate genomes.** Highlighted in light green and beige are the satellite array regions hsat3 and hsat1A, respectively, that include centromeric and centromeric repeats. Stacked barplots show the results for different STR repeating unit lengths.

**
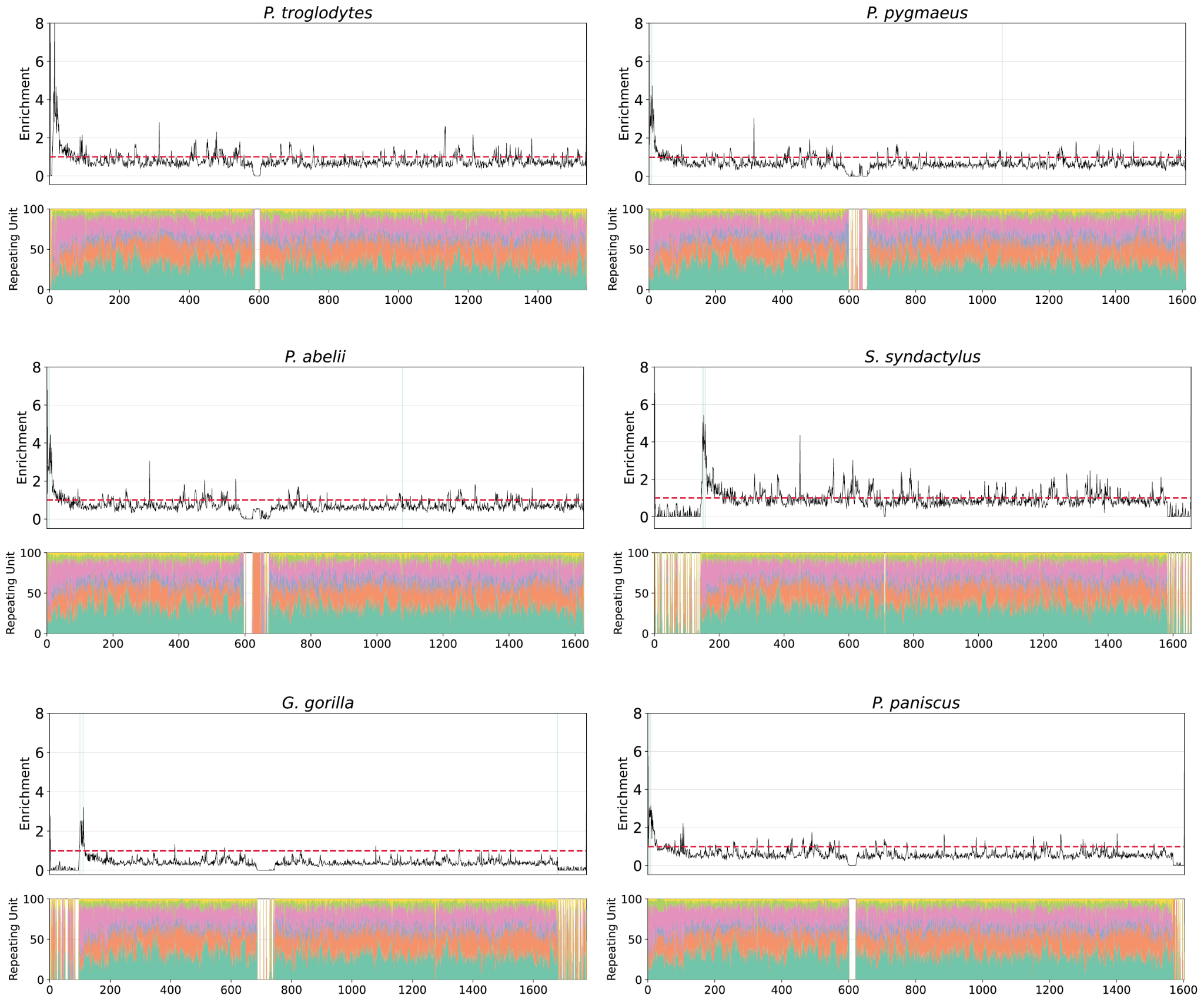
**

**Fig S16: Characterization of STRs across the X chromosomes of the T2T primate genomes.** Stacked barplots show the results for different STR repeating unit lengths.


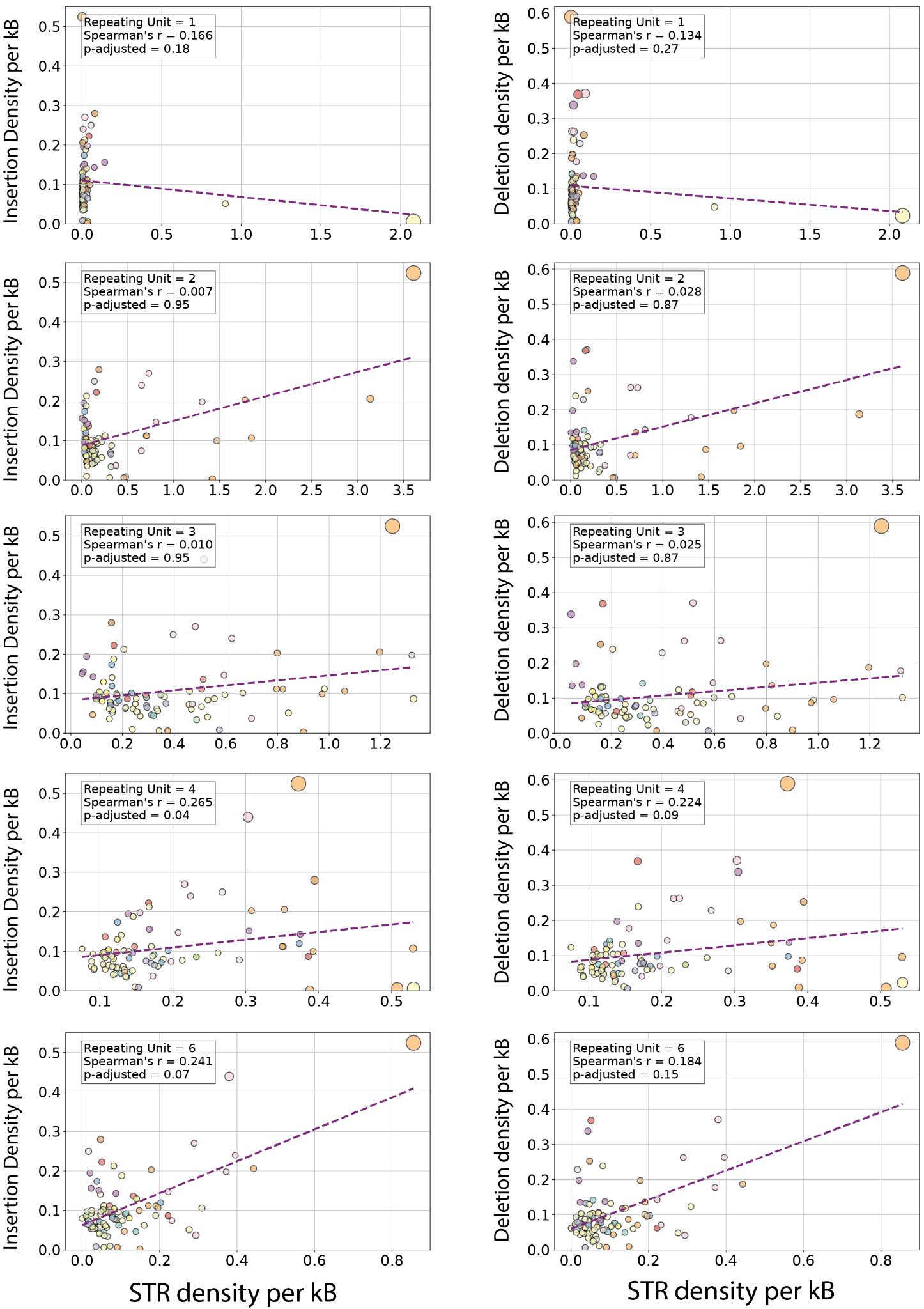


**Fig S17**: **Association of interspecies polymorphic loci with STRs across bacterial organisms.** STR density for repeating units 1, 2, 3, 4, and 6, regressed against small insertions and small deletion polymorphic density for each mutational type across 87 bacterial species, with bubble sizes representing the influence of outliers on the regression as measured by Cook’s distance.

**Table S1: Database breakdown into the three domains of life and viruses.**

| **Domain** | **Total Accessions** | **Total Unique Species** |
| --- | --- | --- |
| Bacteria | 49,192 | 11,119 |
| Viruses | 47,842 | 23,805 |
| Archaea | 687 | 528 |
| Eukaryota | 490 | 291 |

**Table S2: Database breakdown in kingdoms.**

| **Kingdom** | **Total Accessions** | **Total Unique Species** | **Total Phylums** |
| --- | --- | --- | --- |
| Pseudomonadati | 32,297 | 6,529 | 42 |
| Heunggongvirae | 21,286 | 15,247 | 2 |
| Bacillati | 16,337 | 4,316 | 8 |
| Orthornavirae | 9,287 | 3,551 | 6 |
| Bamfordvirae | 6,626 | 395 | 2 |
| Shotokuvirae | 3,168 | 1,247 | 2 |
| Sangervirae | 1,765 | 522 | 1 |
| Methanobacteriati | 495 | 394 | 2 |
| Pararnavirae | 461 | 168 | 1 |
| Fungi | 382 | 213 | 4 |
| Loebvirae | 208 | 148 | 1 |
| Thermoproteati | 164 | 108 | 3 |
| Thermotogati | 156 | 96 | 3 |
| Fusobacteriati | 124 | 36 | 1 |
| Plantae | 50 | 35 | 3 |
| Zilligvirae | 36 | 30 | 1 |
| Protista | 32 | 21 | 4 |
| Metazoa | 26 | 22 | 4 |
| Nanobdellati | 15 | 14 | 5 |
| Trapavirae | 14 | 14 | 1 |
| Abadenavirae | 14 | 14 | 1 |
| Helvetiavirae | 10 | 9 | 1 |
| Promethearchaeti | 5 | 5 | 3 |

**Table S3: Longest, perfect STRs across the genomes studied.** T2T genomes used are marked next to the species names.

| STR Length (bp) | Species name | STR sequence | Phylum | Kingdom |
| --- | --- | --- | --- | --- |
| 235,404 | *Zea mays* (T2T) | *tac* | *Streptophyta* | *Plantae* |
| 175,578 | *Zea mays* (T2T) | *tag* | *Streptophyta* | *Plantae* |
| 154,539 | *Zea mays* (T2T) | *tag* | *Streptophyta* | *Plantae* |
| 130,401 | *Zea mays* (T2T) | *tac* | *Streptophyta* | *Plantae* |
| 102,159 | *Zea mays* (T2T) | *act* | *Streptophyta* | *Plantae* |
| 51,375 | *Solanum tuberosum* (T2T) | *a* | *Streptophyta* | *Plantae* |
| 40,968 | *Zea mays* (T2T) | *agt* | *Streptophyta* | *Plantae* |
| 20,391 | *Ziziphus jujuba* (T2T) | *aacccta* | *Streptophyta* | *Plantae* |
| 17,619 | *Ziziphus jujuba* (T2T) | *gggttta* | *Streptophyta* | *Plantae* |
| 16,744 | *Musa acuminata malaccensis* (T2T) | *tttaggg* | *Streptophyta* | *Plantae* |
| 16,471 | *Glycine max* (T2T) | *ttagggt* | *Streptophyta* | *Plantae* |
| 15,414 | *Aethina tumida* | *taaaatg* | *Anthropoda* | Metazoa |
| 14,166 | *Pan troglodytes* (T2T) | *ttaggg* | *Chordata* | Metazoa |
| 12,725 | *Amyelois transitella* | *ggtta* | *Anthropoda* | Metazoa |
| 11,984 | *Citrus reticulata* (T2T) | *aacccta* | *Streptophyta* | *Plantae* |
| 11,571 | *Vitis vinifera* (T2T) | *ctaaacc* | *Streptophyta* | *Plantae* |
| 11,466 | *Pan troglodytes* (T2T) | *cctaac* | *Chordata* | Metazoa |
| 11,466 | *Symphalangus syndactylus* | *cctaac* | *Chordata* | Metazoa |
| 10,668 | *Citrus x limon* | *tagggtt* | *Streptophyta* | *Plantae* |
| 8,382 | *Pongo pygmaeus* (T2T) | *cctaac* | *Chordata* | Metazoa |
| 8,472 | *Gorilla gorilla* (T2T) | *gggtta* | *Chordata* | Metazoa |
